# Changing the availability and positioning of more vs. less environmentally sustainable products: A randomised controlled trial in an online experimental supermarket

**DOI:** 10.1016/j.appet.2024.107579

**Published:** 2024-06-22

**Authors:** Cinja Jostock, Madison Luick, Susan A. Jebb, Rachel Pechey

**Affiliations:** Nuffield Department of Primary Care Health Sciences, University of Oxford, United Kingdom

**Keywords:** Availability, Positioning, Sustainable food, Choice architecture interventions, Online supermarket, RCT

## Abstract

Food purchasing behaviours are shaped by the choices available to shoppers and the way they are offered for sale. This study tested whether prominent positioning of more sustainable food items online and increasing their relative availability might reduce the environmental impact of foods selected in a 2x2 (availability x position) factorial randomised controlled trial. Participants (n = 1179) selected items in a shopping task in an experimental online supermarket. The availability intervention added lower-impact products to the regular range. The positioning intervention biased product order to give prominence to lower-impact products. The primary outcome was the environmental impact score (ranging from 1 “least impact” to 5 “most impact”, of each item in shopping baskets) analysed using Welch’s ANOVA. Secondary outcomes included interactions (analysed via linear regression) by gender, age group, education, income and meat consumption and we assessed intervention acceptability (using different frames) in a post-experiment questionnaire. Compared to control (mean = 21.6), mean eco quintile score was significantly reduced when availability & order was altered (−2.30; 95%CI: 3.04; −1.56) and when order only was changed (−1.67; 95%CI: 2.42; −0.92). No significant difference between availability only (−0.02; 95%CI: 0.73; 0.69) and control was found. There were no significant interactions between interventions or by demographic characteristics. Both interventions were acceptable under certain frames (positioning emphasising lower-impact products: 70.3% support; increasing lower-impact items: 74.3% support). Prominent positioning of more sustainable products may be an effective strategy to encourage more sustainable food purchasing. Increasing availability of more sustainable products alone did not significantly alter the environment impact of products selected.

## 1 Introduction

Altering dietary behaviour towards consumption of more sustainable foods and drinks could significantly reduce the environmental impact of food systems whilst also having health benefits (Laine et al., 2021; Springmann et al., 2018). An estimated one third of greenhouse gas (GHG) emissions worldwide are attributable to the food system (Crippa et al., 2021), and extensive reductions in GHG emissions are required to avert global warming of 1.5 °C within this century (Intergovernmental Panel on Climate Change, 2021).

To encourage more sustainable eating behaviours interventions are needed to change food purchases. In the UK, the five biggest supermarket chains account for around 75% of the grocery market share (Kantar, 2023) with an estimated 8.5% of all food retail purchases made online in April 2023 (Office for National Statistics, 2023). Decision-making in both physical and online supermarkets tends to be fast (in-store: 9–17 s (Dickson & Sawyer, 1990; Gobb & Hoyer, 1985; Hoyer, 1984; Le Boutillier et al., 1994; Leong, 1993), online: 19 s per product (Anesbury et al., 2016) and choice architecture interventions, which make small modifications to the physical micro-environment, could be influential in shaping behaviour without relying on conscious engagement from individuals (Hollands et al., 2017). Indeed, a meta-analysis using a previously developed choice architecture taxonomy (Münscher et al., 2016) found that such interventions are effective in eliciting food behaviour change (Mertens et al., 2022). However, most research has focused on single interventions whereas in practice a variety of methods are often simultaneously employed.

Examples of choice architecture interventions include changing product placement to emphasise healthier products (Howe et al., 2022; Koutoukidis et al., 2019; Valenčič et al., 2024) and increasing the availability of healthier products in supermarkets (Marty et al., 2020; Piernas et al., 2022), with relatively high public support for both expressed in research from across five countries (Gómez-Donoso et al., 2021). However, evidence on the effectiveness of these interventions in the context of sustainable food choices is relatively limited (Zhuo et al., 2023). We hypothesised that combining availability and positioning interventions may amplify the impact of each individual intervention. For example, if availability works in part through changing social norms regarding the selection of types of products, then positioning these products more prominently may make them more salient and more likely to influence perceived norms (Pechey et al., 2021; Pechey et al., 2020).

Positioning interventions typically increase or decrease the proximity between the individual and the targeted product(s), aiming to elicit a desired response by changing the effort required to obtain an item (Hollands et al., 2019). In online environments, positioning interventions typically list targeted products in salient positions (e.g. top of the page or list in online supermarkets (Howe et al., 2022; Koutoukidis et al., 2019; Valenčič et al., 2024; Zhuo et al., 2023) or online menus (Wyse et al., 2019)). Studies in experimental online supermarkets have found that such prominent positioning of healthier products increased participants’ selection of these products (Howe et al., 2022; Koutoukidis et al., 2019; Valenčič et al., 2024). In contrast, studies investigating more prominent positioning of healthier options in a real-world online canteen ordering system (Wyse et al., 2019) and of more sustainable items in an experimental online supermarket (Zhuo et al., 2023) found no effect, although both interventions involved a limited number of options, with all options visible. The presence of multiple product pages and options may influence the effect due to the higher effort of scrolling, with one study showing that 89% of product choices in online supermarkets stem from the first page (Anesbury et al., 2016).

Availability interventions manipulate the range or frequency of products occurring in an environment (Hollands et al., 2019), and could potentially influence product selections by creating social norms, an increased likelihood of finding the most desired product, or increased visual attention (Pechey et al., 2020). Interventions can change the total number of options (*absolute availability*) or the proportion of certain options within a set of options (*relative availability*), or both (Pechey et al., 2020). A Cochrane review of availability interventions found that reduced availability of targeted foods lowered their selection and consumption, albeit there being low certainty of this effect (Hollands et al., 2019). Indeed, subsequent studies suggest changing the availability of products can increase the selection and purchase of healthier (Marty et al., 2020; Piernas et al., 2022; Reynolds et al., 2021) or more sustainable foods (Garnett et al., 2019; Pechey et al., 2022a) as well as non-alcoholic drinks (Clarke et al., 2023). None of these studies looked at availability and positioning interventions in combination.

This study assessed the effectiveness of availability and positioning interventions, alone and in combination, to encourage the selection of products with a lower environmental impact in an experimental online supermarket platform. We hypothesised that the positioning and availability interventions would interact to reinforce each other because each could increase the salience of and/or ease of acting upon the other. Additionally, potential effect modifiers including demographic characteristics and the differences in the device type used for shopping (given this may affect ease of scrolling and visibility of the full range of options) were explored.

## 2 Methods

### 2.1 Design and setting

This study was a 2x2 factorial randomised controlled trial. The study comprised a screening survey confirming eligibility, a baseline survey assessing participant characteristics, a shopping task, and a post-intervention survey examining the acceptability of the interventions (Appendix A). Surveys were carried out via the online survey platform Qualtrics. The study was conducted in October and November 2022. The study protocol was pre-registered with OSF (https://osf.io/ga4zd).

The shopping task took place in an experimental online supermarket platform (“Woods supermarket”, www.woodssupermarket.co.uk). Departments and aisles in the drop-down menu of the supermarket were ordered to resemble the website of the retailer. Some aisle names were slightly amended and new aisles and shelves were created to make it easier for participants to find products on the shopping list. For example, as burgers were spread out across numerous aisles and shelves, two new aisles/shelves (e.g. “Fresh Meat, Fish, Vegetarian & Vegan Burgers”) were created for fresh and frozen burgers respectively, to raise the probability that participants would be exposed to the interventions. Participants could also use a search bar to look for specific products using keywords. Pages could contain up to 28 products, after which a second page for a shelf is created. There were no budget restrictions and participants were explicitly told that they were not required to pay for items they selected.

Around 8600 products from a major UK grocery retailer were available for selection, and 192 additional low-impact products from five other retailers were added as part of the availability intervention. Products were retrieved from the foodDB database (Harrington et al., 2019), and “ecoscores” were estimated to describe their environmental impact (Clark et al., 2022). These ecoscores, which represent a composite score of greenhouse gas emissions, water scarcity, eutrophication, and land use, fall on a scale of 0–100 and are calculated per 100g of product (Clark et al., 2022) using previously published environmental data (Gephart et al., 2021; Poore & Nemecek, 2018). On the scale, 0 means no impact and 100 is the highest attainable impact (Clark et al., 2022).

Questions in the post-intervention survey (Appendix A.2) assessed acceptability of two types of availability interventions: increasing availability of lower-impact options and decreasing availability of higher-impact options. We expected increasing availability to be more acceptable than decreasing availability as it enlarges choice as opposed to restricting choice (Diepeveen et al., 2013). Additionally, we used two different levels of specification for each question: two were broadly framed as targeting products with “lower-environmental impact” or “higher-environmental impact”, and two specifically mentioned increasing “plant-based and vegetarian” or decreasing “meat, fish and dairy” products. Evidence shows that awareness of the environmental impact of foods is low (Sanchez-Sabate et al., 2019; Sanchez-Sabate & Sabaté, 2019), so that the broadly framed question may not clearly convey what such an intervention may look like in the real-world, potentially impacting acceptability.

### 2.2 Participants and randomisation

Participants were recruited through the research agency Dynata according to the following inclusion criteria: at least 18 years of age, UK resident, able to speak and read English, able to provide informed consent, access to a smartphone or computer and the internet, and the main or shared grocery shopper in their household. Participants were excluded if they followed a vegan or vegetarian diet since the small proportion of the population following these restricted diets could unbalance the randomisation. Participants provided online consent and were equally randomised to one of the four trial arms by Qualtrics, thus achieving allocation concealment. Participant data was anonymous, with only a participant ID given by Dynata.

Participants received the standard panel rate (around £1) for their participation. We encouraged participants to select items they would be willing to consume by offering the opportunity to opt-in for a chance to be one of ten participants to win a randomly selected item from their shopping basket with a value of up to £5. However, for data protection reasons this was not possible. In practice ten randomly selected participants received an additional £5 voucher awarded by Dynata. Participants were informed about this deception at the end of the study, and they had to re-confirm or withdraw their consent. Ethical approval was obtained from the Central University Research Ethics Committee (CUREC) University of Oxford (R65010/RE013).

### 2.3 Shopping task

At the end of the baseline survey, participants received the following instructions:

“You will now be redirected to another website to complete an online shopping task.

**If you are taking this survey on your phone, please rotate your screen into landscape mode**.

We would like you to do some online grocery shopping on a supermarket website. This is not a real online supermarket, and you will not be asked to spend any of your own money.

**We will give you a shopping list and ask you to select a food item to match each item on the shopping list, which will be displayed on the right hand side of the screen**.

**Please do not select additional items**.

When doing your shopping, try to imagine you are doing your own grocery shopping and choose foods that you and your household would eat. You should choose the things you normally buy as far as possible. Do not choose any foods that you would not be willing to eat.”

The following shopping list was displayed in the online supermarket.

- *A pack of burger(s) (meat or veggie)*
- *A pizza*
- *A pie or quiche to share*
- *A ready to eat salad pot*
- *A sandwich, wrap, roll or pasty*
- *A ready meal to heat up*

The four categories of interest were burger (lower-impact items available prior to availability intervention: n = 24); pie or quiche (n = 29); ready meal (n = 159); and sandwich, wrap, roll, or pasty (n = 42).

The pizza and salad categories served as distractors only to provide a more comprehensive shopping list and to reduce the salience of the manipulations.

### 2.4 Interventions

#### 2.4.1 Control

In the control condition, products represented those available in one major UK supermarket and were presented in random order by assigning them a random number between 1.0 and 6.0 (e.g. 1.029926; 4.494886) and ordering accordingly. No additional lower-environmental impact products were added to the website.

#### 2.4.2 Availability only

In the availability condition, products present in the control group kept the same order value. Lower-environmental impact options were added for the four food categories of interest only. These equated to adding all products with an ecoscore in the lowest two quintiles (quintiles by environmental impact, explained below), available from 5 other UK supermarkets, except own-brand products. The proportion of lower impact options was raised from between 22 and 35% to between 37 and 52% of categories. These products were assigned a newly generated random order value determining their position on the website, using the same method as for the control group. No higher-impact products were removed.

#### 2.4.3 Order only

In the order condition, only products of interest were ordered with a bias towards more sustainable options, increasing the probability that these products appear on earlier pages (Table 1 shows the percentage shown on the first page for each shopping list item by group). The eco quintile of products determined the range in which the new order value would fall.

- Eco quintile of 1 (lowest environmental impact): random value between 1 and 2,
- Eco quintile of 2: random value between 1.1 and 3,
- Eco quintile of 3: random value between 1.2 and 4,
- Eco quintile of 4: random value between 1.3 and 5,
- Eco quintile of 5 (highest environmental impact): random value between 1.4 and 6.

All other products had the same order value as in the control condition. No additional products were added.

#### 2.4.4 Availability & order

**Fig. 1:**
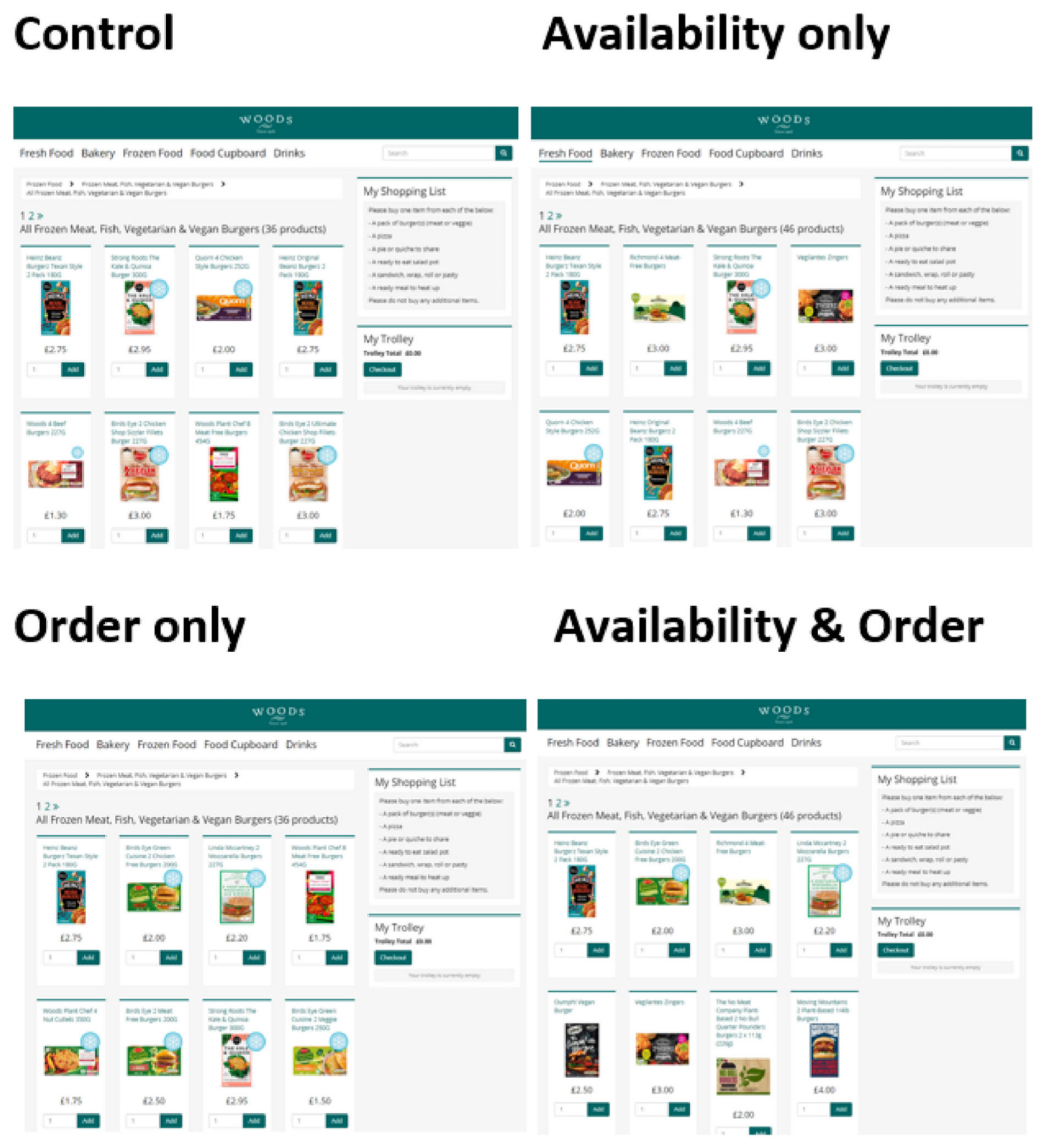
Top of the first page of the frozen burger shelf for each condition.

Here, the full range of products included in the availability intervention were offered in an order that favoured the more sustainable. The additional products were assigned an order value according to the same methods used in the order only condition. Fig. 1 shows an example of the top of the page for one of the burger shelves. Further examples of online supermarket pages for the other shopping list items of interest are in Appendix B.

### 2.5 Sample size

Based on a small effect size (f = 0.1) for any interaction effect between the order and availability interventions on environmental impact score, and with 90% power and alpha of 0.05, the required sample size was 1053. We aimed to achieve 290 participants per group, to account for an estimated 10% of missing data.

### 2.6 Statistical analysis

Participants who bought less than 6 or more than 9 items as well as speeders, who completed the study in less than 30% of the median time, were excluded from analysis. The main analysis only included participants who bought products from at least 5 different categories on the shopping list and 6 to 9 items in total (i.e. sufficient to have bought one product for each shopping list item, but allowing for the possibility of buying more than one pack for some shopping list items).

#### 2.6.1 Primary analysis

The primary outcome was planned to be the difference in total ecoscores of shopping baskets (containing all products that participants selected in the supermarket) between the four trial arms. However, first inspections of the data revealed that the ecoscore variable had a bimodal distribution. This was caused by the burger category, for which scores were either relatively low (e.g. plant-based burgers) or high (e.g. beef burgers). A boxplot showing the summed ecoscores of participant’s shopping baskets per group and a Tukey test (following a significant ANOVA test result (p=<0.001)) investigating group differences in total ecoscore of baskets with burgers removed are included in Appendix C.

Consequently, in a deviation from the pre-specified statistical analysis plan, eco quintile scores were used in place of ecoscores. Eco quintile scores of products were summed across shopping baskets of participants to compare total eco quintile scores between groups. The mean eco quintile scores of shopping baskets were compared using a Welch ANOVA (due to unequal variance), followed by a Games-Howell post-hoc test.

##### Interaction analysis

A two-way ANOVA was used to test for an interaction effect between the two interventions.

##### Sensitivity analysis

The sensitivity analysis included only participants who complied 100% with the shopping list (i.e., bought exactly six items and one product for each category on the shopping list).

Analyses applied a threshold of p ≤ 0.05 to determine statistical significance.

#### 2.6.2 Secondary analyses

For all secondary and exploratory outcomes that planned to use ecoscores of baskets as outcomes, eco quintile scores were used instead. Secondary analyses applied a threshold of p ≤ 0.003 (Bonferroni adjustment) to determine statistical significance.

##### Individual environmental indicators

Individual environmental indicator quintile scores (i.e. greenhouse gas emissions, water scarcity, land use, and eutrophication) of shopping baskets were compared between groups using simple linear regression. The summed environmental indicator scores also had a bimodal distribution and were thus assigned a quintile score ranging from 1 to 5 based on quintiles according to the same methods as used to produce the eco quintile scores.

##### Interactions by demographic characteristics or device type

Interactions for covariates (group, age group, gender, education, income, meat consumption, and device broadly separated by Qualtrics into tablets and mobiles and others such as computers; see Appendix A.1 for baseline survey) were tested using multiple linear regression, investigating each interaction in a separate model. Details on demographic characteristics and their categorisation are summarised in Table 2.

#### 2.6.3 Exploratory analyses

##### Variation by shopping list item

Two sets of analyses were carried out to explore potential differences within food categories (see Appendices D and E for more details).

1. The effect of trial group on the proportion of products selected that were in (1) the lowest 40% (i.e. eco quintiles 1 & 2) and (2) the highest 40% (i.e. eco quintiles 4 & 5) of products in terms of environmental impact was investigated using logistic regression models.
2. The intervention effects per shopping list item were investigated through chi-square tests and descriptive statistics, creating bar graphs showing the proportion of products in each eco quintile by group.

##### First page placement

Pre-specified multi-level regression was replaced by logistic regression models for each shopping list item of interest for this outcome, assessing the impact of the percentage of lower-impact products (eco quintile 1 or 2) on the first page on the selection of these products. For these analyses, mean eco quintile score of selected products and mean percentage of lower-impact products of shelves were used if participants selected more than one product of the same category.

##### Acceptability of interventions

Acceptability was measured on a 7-point scale (“strongly support” to “strongly oppose”) (Appendix A.2) and evaluated using descriptive statistics.

##### Basket cost

Basket prices (£GBP) were compared using descriptive statistics and a Welch ANOVA due to unequal variance.

Due to several tests being conducted, exploratory analyses used the same significance threshold as secondary analyses (p ≤ 0.003).

The research team member analysing the primary outcome was blinded to intervention allocation. All analyses were carried out using R (Version 4.1.3.) (R Core Team, 2022) and RStudio, using the dplyr (Wickham et al., 2022), tidyr (Wickham & Girlich, 2022), rstatix (Kassambara, 2022), car (Fox & Weisberg, 2019), and ggplot2 (Wickham, 2016) packages.

## 3 Results

**Fig. 2:**
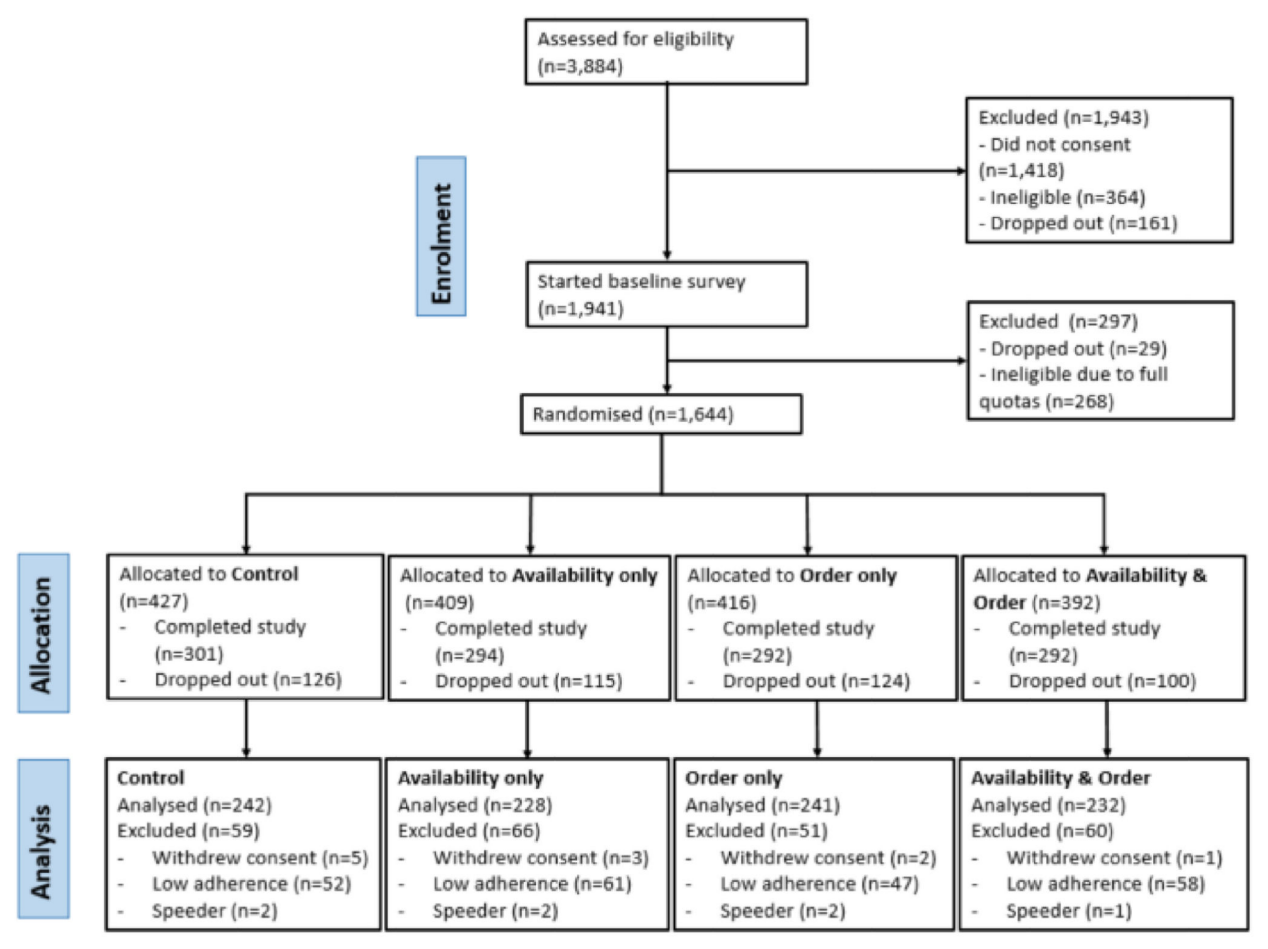
CONSORT flow diagram (Schulz et al., 2010). Note: After the first ~100 participants, online supermarket settings were set to only allow participants to check out if their shopping baskets contained between 6 and 15 different items to prevent checkouts with empty baskets. Although Qualtrics randomised participants equally, continuous monitoring of completion numbers revealed that some groups had slightly higher rates of participants not completing the whole survey (marked as “dropped out” in the figure). Therefore, randomisation counts on Qualtrics were adjusted around the mid-point of recruitment to achieve approximately the same sample size in each group.

Out of 1179 participants who completed the study, 943 complied with at least 75% of items on the list and were included in the main analysis (Fig. 2).

The mean age of eligible participants was 46.6 years (range: 18-84y) with median household size of 2 (range: 1–10) and reported mean food shopping spend of £82 per week. A third of participants (n = 338; 35.5%) had not ordered groceries online in the last year, and 113 (12%) ordered groceries online once per week or more. Participants selected a mean of 6.1 products and spent a median of 11.1 min completing the study. There were no significant differences in gender, age group, education, income, meat consumption, device type used, household size, weekly shopping expenses, or quantity of products selected between the groups (Table 2).

In a post experiment survey, 43.3% (n = 408) of participants either somewhat or strongly agreed that they often think about the environmental impact of the food items they select when shopping, whilst 30.9% (n = 291) of participants either somewhat or strongly disagreed (Table 2).

### 3.1 Primary outcomes

**Fig. 3:**
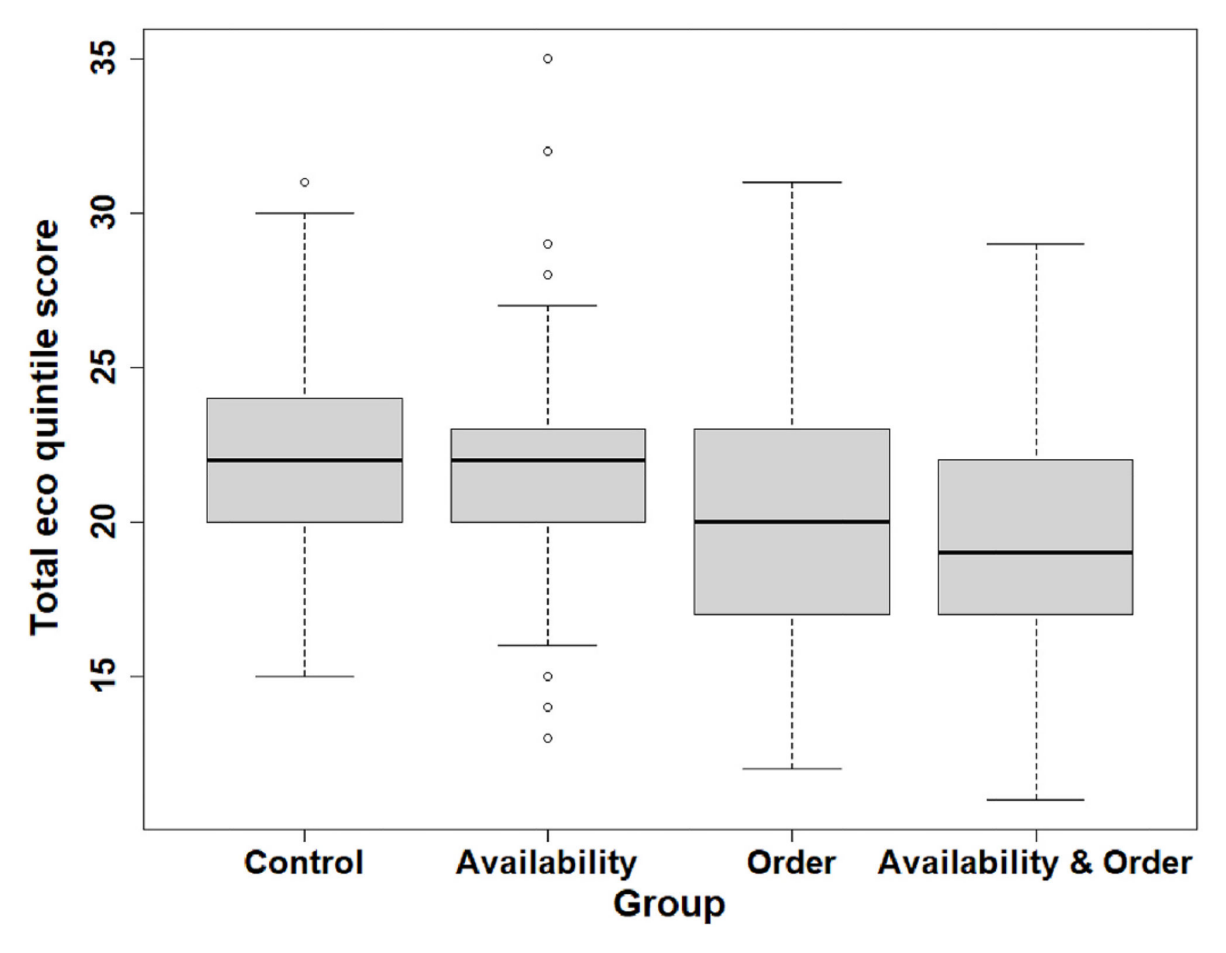
Total eco quintile score of participant’s shopping baskets by group.

The mean summed eco quintile score of shopping baskets of groups was highest for the control condition (21.6) and availability only (21.6), followed by order only (19.9) and availability & order (19.3) (Fig. 3).

All group differences were significant apart from control vs. availability only, and order only vs. availability & order groups (Table 3). Compared to the control group, the availability & order intervention resulted in the largest decrease in mean summed eco quintile score (−2.30; 95% CI: 3.04; −1.56), followed by the order only group (−1.67; 95% CI: 2.42; −0.92). There was no significant interaction between the two interventions (p = 0.15).

The sensitivity analysis including only participants who fully complied with the shopping list (662 participants) showed results similar to the main analysis: the order only and availability & order groups had significantly reduced mean summed eco quintiles scores of shopping baskets compared to availability only and control, but no significant differences were found for availability only vs. control and availability & order vs. order only (Appendix F).

### 3.2 Secondary outcomes

#### Individual environmental indicators

For all four environmental indicators, mean eco quintile indicator scores were significantly lower for the order only and availability & order groups compared to the control group, with the availability & order group recording the largest reductions (Appendix G). No significant differences were found between the control and availability only groups for any of the indicators.

#### Interactions by demographic characteristics

The effect of the interventions did not differ significantly by gender, age, education, income, or meat consumption (supplementary file 1). The interaction model for gender excluded participants identifying as another gender due to very small group size (n = 3).

#### Interactions by device type

The effect of the intervention was similar whether the study was performed on a mobile or tablet or other such as computer (supplementary file 1; final model without interaction terms in Appendix H).

### 3.3 Exploratory outcomes

#### Variation by shopping list item

Analyses per shopping list item of interest were broadly in line with the primary results, showing that the pattern of product choices was similar across shopping list items (see Appendices D and E).

#### First page placement

More sustainable items were significantly more likely to be selected if there was a greater proportion of more sustainable products on the first page, with a one unit increase in percentage raising the odds of choosing a lower-impact product by 2% (burgers), 3% (pies), 4% (sandwiches), or 5% (ready meals) (Table 4). Additionally, exploratory analyses showed that the majority of pizzas and salads (distractor items) chosen by participants were on the first page (88% for salad, 81% for pizza).

#### Acceptability of interventions

**Fig. 4:**
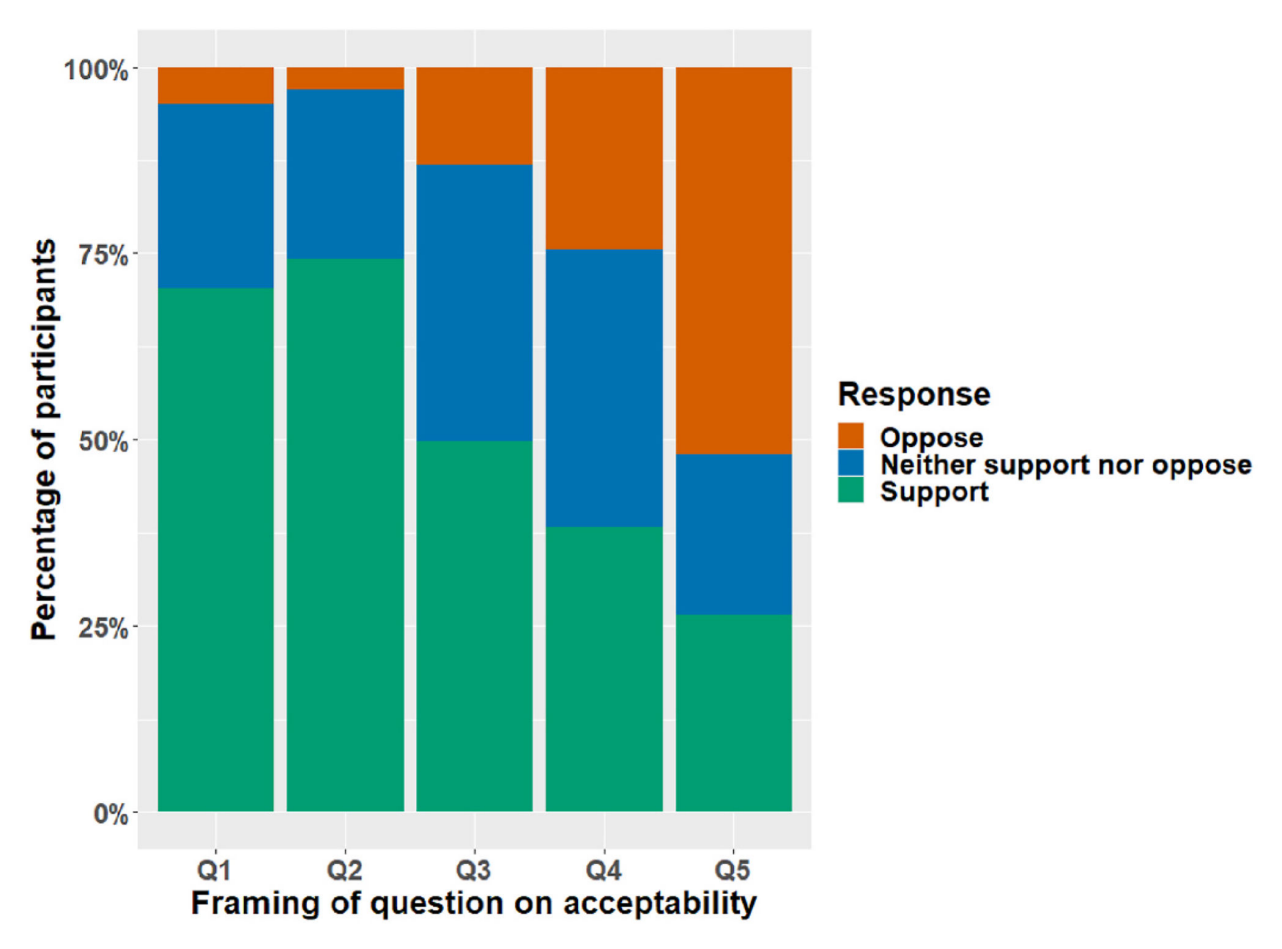
Acceptability of the interventions. Note: Participants were asked ‘*to what extent would you support or oppose’* Q1: *“If supermarkets were to introduce a feature that positioned products to emphasise products with a lower-environmental impact?”* (n=943); Q2: *“If supermarkets were to offer a greater range of products with a lower-environmental impact?”* (n=940); Q3: *“If supermarkets were to offer a greater range of plant-based and vegetarian products?”* (n=942); Q4: *“If supermarkets were to offer a smaller range of products with a higher-environmental impact?”* (n=941); Q5: *“If supermarkets were to offer a smaller range of meat, fish and dairy products?”* (n = 943). For more details, see additional file 9.

The majority of respondents were supportive of lower environmental impact products having a more prominent position and only few participants opposed such a feature (Fig. 4; Appendix I, Fig. I1). Acceptability of interventions to increase low impact products varied according to the framing of this intervention (Fig. 4; Appendix I, Fig. I2-I5). Support was high for offering more products with a lower environmental impact, but markedly lower when specifically asking whether respondents would support introducing a greater range of plant-based and vegetarian products. Scenarios which involved restricting availability of higher environmental impact attracted significant opposition, especially where this involved offering a smaller range of meat, fish and dairy products.

#### Basket cost

The mean basket price was similar in all conditions (p = 0.05) (Appendix J).

## 4 Discussion

Biasing the product order listing to favour more sustainable products significantly reduced the environmental impact of selected products in an experimental online supermarket shopping task. We found no evidence of a difference in the sustainability of product selections when increasing the availability of lower-impact options. Results were consistent for individual environmental indicators, and the pattern of results for the positioning and combined interventions was similar across all four food categories targeted.

The main strengths of this study include the randomised design which helps to offset the limitations of a virtual design to identify the relative effectiveness of different interventions. The virtual online store closely mimicked a typical UK online shop, with product ranges in line with actual availability in online supermarkets. Key limitations include that firstly, participants did not receive any actual products and did not spend any money, potentially influencing their product selection. To minimise this risk, participants were told ten randomly selected participants would receive one randomly selected product from their shopping basket. Secondly, the proportion of participants fully adhering to the shopping task was lower than expected. However, the main analysis and sensitivity analysis concurred. Thirdly, around a third of participants indicated they had not purchased groceries online in the past year - while this should not impact on our comparisons between groups, given the randomised design, it is possible that the effect size for online shopping may differ if non-frequent online shoppers behave differently to regular online shoppers. Lastly, the planned outcome variable was changed due to bimodality. It is important to note that the range of ecoscores within eco quintile categories increases as the eco quintile category becomes higher (i.e., is lowest for eco quintile 1 and highest for eco quintile 5). For example, switching from a beef burger with an ecoscore of 44 (eco quintile 5) to a plant-based burger with an ecoscore of 1.4 (eco quintile 2) represents a bigger change in environmental impact than a change from a duck wrap with an ecoscore of 3.98 (eco quintile 4) to a bean wrap with an ecoscore of 1.01 (eco quintile 1). Nevertheless, our analysis of eco quintile scores gives a good indication of potential behaviour change.

The findings of a significant effect of the positioning intervention are in line with a previous study to encourage purchasing of items containing less saturated fat (Koutoukidis et al., 2019). The positioning intervention used in this study was less extreme as it included a random component in the ordering of products instead of ordering according to the outcome of interest only. Yet, there was still a significant effect, supporting the effectiveness of online positioning interventions even at lower strengths. The random component is important because real-world online supermarkets may be hesitant to order products only according to environmental impact, whilst considering sustainability as one of the factors determining the order of products may be more acceptable. Additionally, participants rated the positioning intervention as very acceptable.

In contrast, other online studies did not find an effect of changing the order of products on the healthiness (Wyse et al., 2019) or sustainability (Zhuo et al., 2023) of product choices. However, all products were shown on one page, which removes the potential impact of first vs. later page placement. Our exploratory analyses highlighted the importance of first page placement in an RCT with a large sample size, adding to Anesbury et al. (2016) observational study with a smaller sample (n = 40) in a real-world online supermarket. Previous research has also shown that only few online shoppers changed the default order or number of products shown per page (Anesbury et al., 2016), suggesting that interventions targeting default settings would reach most shoppers whilst maintaining choice. Future research should further investigate these potential mechanisms behind positioning interventions, preferably through RCTs in real-world online settings, to optimise their effectiveness. Effects of positioning interventions in real-world online supermarkets may be smaller as customers may use website features such as pre-existing baskets or scrolling through previously bought products, or adding all ingredients for a retailer-suggested recipe through one click. A study moving healthier products to higher positions on a page in a real-world online supermarket found no evidence of an effect, however the sample size may have been insufficient to detect meaningful effects (Bunten et al., 2022). Robust studies of positioning interventions in real-world online supermarkets are needed.

We found no significant difference in the environmental impact of selected products between control and the availability intervention in our study. Previous studies have found availability interventions to be effective (Hollands et al., 2019; Marty et al., 2020). Existing evidence mostly comes from studies in non-supermarket settings such as cafeterias or vending machines, involving a limited set of options (Garnett et al., 2019; Hollands et al., 2019; Pechey et al., 2022a; Reynolds et al., 2021). If numerous options are already available, there may be a ceiling effect or a larger proportional change may be required. In online supermarket contexts, two previous studies found effects when partially removing less healthy items (Marty et al., 2020) or alcoholic drinks (Clarke et al., 2023), with greater proportional change (33%–67% and 25%–75% vs. 22–35% to 37–52%). Indeed, when the proportion of non-alcoholic drinks increased from 25% to 50% there was no significant differences in alcohol units selected (Clarke et al., 2023). We focused on increasing the absolute availability of low-impact products and it is plausible that removing higher-impact products would be more effective. Moreover, this paper supports the findings of Anesbury et al. (2016), suggesting that first page positioning is key. As such, the availability on the first page – rather than availability across the whole range – may be a more important marker to consider for public health interventions. However, availability interventions can take many different forms, and whilst we find no evidence of an effect of an availability intervention in our study, other operationalisations of availability interventions (e.g. also removing less sustainable options) should be tested.

In line with our expectations, increasing availability was perceived as more acceptable than decreasing availability. This is consistent with prior research on the effect of framing on the acceptability of nudges, which shows that more intrusive interventions (e.g. restrictions) are less acceptable than those that are less intrusive but may require more agency (e.g. providing information or enabling choice) (Diepeveen et al., 2013; Jung & Mellers, 2016). Decreasing availability restricts options while increasing availability expands options. Previous research has suggested low acceptability of policies to reduce meat availability (Pechey et al, 2022b). Evidence demonstrates a lack of awareness of the environmental harms of meat and consumers do not perceive lowering meat consumption as an effective strategy to combat climate change (Sanchez-Sabate et al., 2019; Sanchez-Sabate & Sabaté, 2019), which may partially explain the different patterns of acceptability between questions. Furthermore, participants may perceive the targeting of specific products as unfair (and therefore less acceptable (Bergquist et al., 2022)) compared to targeting all lower- or higher-impact products. Nonetheless, in a large-scale effort to make their food stores more sustainable, food retailer Lidl has declared their intention to reduce the assortment of meat products whilst expanding the range of plant-based options (Buxton, 2023).

We found no significant interactions by demographic characteristics or device type, meaning that these interventions, where effective, could contribute to population-level shifts in dietary behaviour without increasing inequalities. There were no differences in mean price of baskets across intervention groups, underscoring the economic viability of these interventions for stores. Future research should investigate whether these effects translate to real-world online supermarkets as well as other online food settings.

## 5 Conclusion

This study provides evidence for the effectiveness of more prominent positioning, though not increased availability, of lower environmental impact foods on an online supermarket website to encourage selection of more sustainable items. Both interventions were rated as acceptable and showed no evidence for differential effects across socioeconomic groups. Future research should assess how positioning interventions perform in real-world online supermarkets.

## Supplementary Material

**Appendix A. Supplementary data** Supplementary data to this article can be found online at https://doi.org/10.1016/j.appet.2024.107579.

Supplementary information

Supplementary information

## Figures and Tables

**Table 1:** Percentage of lower-impact products (eco quintile 1 or 2) on the first page of key shelves for shopping list items.

Group		Control		Availability only		Order only		Availability & Order	
**Shopping list item & key shelves**									
**Burgers**									
*All Fresh Meat, Fish, Vegetarian & Vegan Burgers*		21.4		39.3		28.6		53.6		
*All Frozen Meat, Fish, Vegetarian & Vegan Burgers*		35.7		53.6		57.1		75		
**Sandwiches**										
*Sandwiches & Wraps*		14.3		17.9		25		28.6		
*Pasties & Snacking*		25		42.9		57.1		75		
*All Frozen Sausage Rolls, Pasties & Snacks*		57.1		71.4		67.9		89.3		
**Pies**										
*Pies & Quiches*		21.4		35.7		39.3		60.7		
*All Frozen Pies*		35.7		50		35.7		57.1		
**Ready meals**										
*Ready Meals*		10.7		14.3		39.3		42.9		
*Frozen Ready Meals*		21.4		28.6		60.7		75		
*All Tinned & Packaged Ready Meals*		39.3		57.1		71.4		82.1		

For sandwiches, the shelves ‘Pasties & Snacking’ and ‘All Frozen Sausage Rolls, Pasties & Snacks’ also contained products not qualifying as part of the sandwich category.

**Table 2:** Demographic characteristics of participants.

		Control		Availability		Order		Availability & Order		Chi-square or Kruskal-Wallis test (p-value)
		(n = 242) (%)		(n = 228) (%)		(n = 241) (%)		(n = 232) (%)		
**Gender**										0.63**^a^**
*Female*		122 (50.4)		118 (51.8)		135 (56)		125 (53.9)		
*Male*		119 (49.2)		110 (48.2)		106 (44)		105 (45.3)		
*Identify as another gender*		1 (0.4)		/		/		2 (0.9)		
**Age group**										0.55
*18-24*		31 (12.8)		35 (15.4)		32 (13.3)		30 (12.9)		
*25-34*		49 (20.2)		34 (14.9)		45 (18.7)		40 (17.2)		
*35-44*		37 (15.3)		37 (16.2)		29 (12)		42 (18.1)		
*45-54*		35 (14.5)		36 (15.8)		50 (20.7)		41 (17.7)		
*55-64*		33 (13.6)		44 (19.3)		34 (14.1)		34 (14.7)		
*65+*		57 (23.6)		42 (18.4)		51 (21.2)		45 (19.4)		
**Education^b^**										0.85
*Up to 4 GCSE’s*		30 (12.4)		23 (10.1)		25 (10.4)		23 (9.9)		
*5 or more GCSE’s or 1 A-level*		29 (12)		42 (18.4)		40 (16.6)		34 (14.7)		
*2 or more A-levels*		59 (24.4)		53 (23.2)		52 (21.6)		58 (25)		
*Bachelor’s degree*		77 (31.8)		72 (31.6)		86 (35.7)		80 (34.5)		
*Postgraduate degree*		47 (19.4)		38 (16.7)		38 (16)		37 (16)		
**Income**										0.95
*Below £15.5k*		40 (16.5)		28 (12.3)		39 (16.2)		36 (15.5)		
*Between £15.5k up to and including £25k*		39 (16.1)		42 (18.4)		39 (16.2)		38 (16.4)		
*Between £25k and £39k*		62 (25.6)		53 (23.2)		61 (25.3)		51 (22)		
*£40k or above*		88 (36.4)		92 (40.4)		93 (38.6)		93 (40.1)		
*Prefer not to say*		13 (5.4)		13 (5.7)		9 (3.7)		14 (6)		
**Meat consumption^c^**										0.55
*Low*		84 (34.7)		90 (39.5)		100 (41.5)		90 (38.8)		
*Medium*		97 (40.1)		75 (32.9)		88 (36.5)		82 (35.2)		
*High*		60 (24.8)		61 (26.8)		51 (21.2)		59 (25.4)		
*NA*		1 (0.4)		2 (0.9)		2 (0.8)		1 (0.4)		
**Device used for study**										0.38
*Desktop/Laptop*		145 (59.9)		120 (52.6)		140 (58.1)		127 (54.7)		
*Mobile/Tablet*		97 (40.1)		108 (47.4)		101 (41.9)		105 (45.3)		
**Median household size**		2		2		2		2		0.07
**Participants selecting 6 products**		227 (93.8)		206 (90.4)		221 (91.7)		215 (92.7)		0.55
**Median weekly shopping expenses (£)**		70		70		70		70		0.69
**Online shopping frequency**										/
*Once* per *week or more often*		27 (11.2)		27 (11.8)		31 (12.9)		28 (12.1)		
*1–3 times* per *month*		33 (13.6)		32 (14)		32 (13.3)		34 (14.7)		
*4–11 times in the past year*		56 (23.1)		34 (14.9)		39 (16.2)		36 (15.5)		
*1–3 times in the last year*		52 (21.5)		41 (18)		54 (22.4)		48 (20.7)		
*Never or not in the last year*		74 (30.6)		94 (41.2)		85 (35.3)		85 (36.6)		
*Prefer not to say*		/		/		/		1 (0.4)		
**Often think of environmental impact whilst shopping**										/
*Strongly agree*		12 (5)		12 (5.3)		12 (5)		19 (8.2)		
*Somewhat agree*		97 (40.1)		87 (38.2)		85 (35.3)		84 (36.2)		
*Indifferent*		66 (27.3)		57 (25)		63 (26.1)		58 (25)		
*Somewhat disagree*		33 (13.6)		44 (19.3)		46 (19.1)		45 (19.4)		
*Strongly disagree*		34 (14)		28 (12.3)		35 (14.5)		26 (11.2)		

a Chi-square tests for gender excluded participants identifying as another gender due to very small group size (n = 3).

b Education categories adapted from UK census categories (Office for National Statistics, 2013) (see Appendix G for category details).

c Meat consumption: Participants were asked in three separate questions how often per week they consume meat for a. breakfast, b. lunch, c. dinner. Answers to each of the three questions received a score: 0 for “Less than once a week”, 1 for “1–2 days a week”, 2 for “3–4 days a week”, 3 for “5–6 days a week”, 4 for “Every day”. Scores were then summed to obtain an overall meat consumption score, categorised as low (score between 0 and 4), medium (score between 5 and 8) or high (score between 9 and 12).

**Table 3:** Total environmental impact of shopping baskets between groups.

Group 1	Group 2	Estimate	Conf. low	Conf. high	p-value
**C**	A	–0.02	–0.73	0.69	1
**C**	O	–1.67	– 2.42	– 0.92	*<*0.001 (*)
**C**	AO	– 2.30	– 3.04	–1.56	*<*0.001 (*)
**A**	O	–1.65	– 2.45	– 0.85	*<*0.001 (*)
**A**	AO	– 2.28	– 3.07	–1.49	*<*0.001 (*)
**O**	AO	– 0.63	–1.46	0.2	0.20

Note.

* denotes significance at p ≤ 0.05.

**Table 4:** Impact of the percentage of lower-impact products on the first page on the selection of lower-impact products.

	OR	Lower 95% CI	Upper 95% CI	Estimate	p-value	Lower 95% CI	Upper 95% CI
***Burger***				*–2.33*		*– 2.84*	*–1.84*
	*1.02*	*1.01*	*1.03*	*0.02*	<0.001 (*)	*0.01*	*0.03*
***Sandwich***				*– 2.33*		*– 2.71*	*–1.97*
	*1.04*	*1.03*	*1.05*	*0.04*	<0.001 (*)	*0.02*	*0.05*
***Pie***				*– 2.33*		*– 2.82*	*–1.85*
	*1.03*	*1.02*	*1.04*	*0.03*	<0.001 (*)	*0.02*	*0.04*
***Ready meal***				*– 3.23*		*– 3.71*	*– 2.80*
	*1.05*	*1.04*	*1.06*	*0.05*	<0.001 (*)	*0.04*	*0.06*

*Note*. Significance threshold of p ≤ 0.003. Number of participants: burgers = 909 [C: 236; A: 220; O: 232; AO: 221], sandwiches = 857 [C: 218; A: 203; O: 223; AO: 213], pies = 927 [C: 238; A: 220; O: 238; AO: 229], ready meals = 915 [C: 236; A: 220; O: 235; AO: 224]. Mean eco quintile score and mean proportion of shelves was used to account for some participants choosing more than one item per shopping list category.

## Data Availability

The dataset(s) supporting the conclusions of this article are available in the OSF repository https://osf.io/xu5pt/.
