# Supplementary material for: Changing the availability and positioning of more vs. less environmentally sustainable products: A randomised controlled trial in an online experimental supermarket
Source: Appetite. Author manuscript; Available in PMC 2025 Nov 28. (PMC7618412; doi:10.1016/j.appet.2024.107579)
Supplement: Supplementary information [file EMS211093-supplement-Supplementary_information.docx]

**Appendices**

Contents

[Appendix A. Baseline survey & acceptability questions (post-intervention survey) 2](#_Toc166488141)

[Appendix A.1: Baseline survey 2](#_Toc166488142)

[Appendix A.2: Acceptability questions in post-intervention survey 3](#_Toc166488143)

[Appendix B: Examples of product pages in the online supermarket 5](#_Toc166488144)

[Figure B1. Sandwiches: Pasties & Snacking 5](#_Toc166488145)

[Figure B2. Pies: All Frozen Pies 5](#_Toc166488146)

[Figure B3. Ready meals: All Ready Meals 6](#_Toc166488147)

[Appendix C: Boxplot of originally planned outcome measure (ecosores) by group and analysis with burger category removed 7](#_Toc166488148)

[Figure C. Total Ecoscores of participants’ shopping baskets by group 7](#_Toc166488149)

[Table C. Total environmental impact (original Ecoscore measure) of shopping baskets between groups, with burger category removed 7](#_Toc166488150)

[Appendix D: Difference in proportion of low- and high-impact products selected by group (secondary outcome) 7](#_Toc166488151)

[Table D1: Choice of shopping list items of interest in lowest 40% of environmental impact by group 8](#_Toc166488152)

[Table D2: Odds ratio and percentage of participants choosing a lower-impact item 9](#_Toc166488153)

[Table D3: Choice of shopping list items of interest in highest 40% of environmental impact by group 9](#_Toc166488154)

[Table D4: Odds ratio and percentage of participants choosing a higher-impact item 10](#_Toc166488155)

[Appendix E: Effect of interventions within food categories 10](#_Toc166488156)

[Appendix F: Sensitivity analysis 12](#_Toc166488157)

[Appendix G: Analysis of individual environmental indicators 13](#_Toc166488158)

[Appendix H: Total Eco quintile score of participant’s shopping baskets by group, demographic characteristics and device type used for shopping online 13](#_Toc166488159)

[Appendix I: Acceptability of the interventions 15](#_Toc166488160)

[Figure I1. Acceptability of the positioning intervention 15](#_Toc166488161)

[Figure I2. Acceptability of increasing the availability of lower-impact products 16](#_Toc166488162)

[Figure I3. Acceptability of increasing the availability of vegetarian and plant-based products 16](#_Toc166488163)

[Figure I4. Acceptability of decreasing the availability of higher-impact products 17](#_Toc166488164)

[Figure I5. Acceptability of decreasing the availability of meat, fish and dairy products 18](#_Toc166488165)

[Appendix J: Welch ANOVA for basket price 18](#_Toc166488166)

[References 18](#_Toc166488167)

# Appendix A. Baseline survey & acceptability questions (post-intervention survey)

## Appendix A.1: Baseline survey

1. Demographic characteristics

- Please indicate what gender you identify with
  - Male
  - Female
  - I identify as another gender
- Please tell us your age (in years)
  - [FREE TEXT ANSWER]

1. Please indicate your household income (total household income before tax)

- Below £15.5K
- Between £15.5K up to and including £25K
- Between £25K and £39K
- £40K or above
- Prefer not to say

1. Household size – “How many people live at your house, including you?
   - **Please specify** (in numbers)
     - [FREE TEXT ANSWER]
   - Prefer not to say
2. Highest educational level) - “What is the highest education qualification you have achieved?” (categories based on UK census categories ^(1)^)

- None
- Up to 4 GCSE's (Including 1-4 O Levels/CSE/GCSEs (any grades), Foundation Diploma, NVQ level 1, Foundation GNVQ or equivalents)
- 5 or more GCSE's or 1 A-level (Including 5+ GCSEs (Grades A*-C),1 A Level/ 2-3 AS Levels, NVQ level 2, Intermediate GNVQ, City and Guilds Craft, BTEC First/General Diploma, RSA Diploma, Apprenticeship or equivalents)
- 2 or more A-levels (Including 2+ A Levels, 4+ AS Levels, NVQ Level 3, Advanced GNVQ, City and Guilds Advanced Craft, ONC, OND, BTEC National, RSA Advanced Diploma or equivalents)
- Bachelor's degree (Including BA, BSc, NVQ Level 4-5, HNC, HND, RSA Higher Diploma, BTEC Higher level or equivalents)
- Post-Graduate degree or qualification (Including Higher Degrees e.g. MA, PhD, PGCE, Professional qualifications e.g. teaching, nursing, accountancy or equivalents)

1. Regular shopping

“On average, how much do you spend on supermarket shopping per week (in £)?”

- - **Please specify** (in numbers)
    - [FREE TEXT ANSWER]
  - Prefer not to say

1. Online shopping experience

“How often, on average over the past year, have you shopped online for food or groceries to be delivered to you (e.g. Tesco.com, Ocado.com, mysupermarket.co.uk)?“

- Never or not in the last year
- 1-3 times in the last year
- 4-11 times in the last year
- 1-3 per month
- Once per week or more often.
- Prefer not to say

1. How many times a week do you typically eat meat at breakfast/lunch/dinner?

|  | Less than once a week | 1-2 days a week | 3-4 days a week | 5-6 days a week | Every day | Prefer not to say |
| --- | --- | --- | --- | --- | --- | --- |
| Breakfast |  |  |  |  |  |  |
| Lunch |  |  |  |  |  |  |
| Dinner |  |  |  |  |  |  |

## Appendix A.2: Acceptability questions in post-intervention survey

1. Do you often think about the environmental impact of the foods you select when doing your shopping?

- Strongly agree
- Somewhat agree
- Indifferent
- Somewhat disagree
- Strongly disagree

1. “If supermarkets were to introduce a feature that positioned products to emphasise products with a lower-environmental impact, to what extent would you support or oppose this?

- Strongly support
- Support
- Somewhat support
- Neither support nor oppose
- Somewhat oppose
- Oppose
- Strongly oppose

1. “If supermarkets were to offer a greater range of products with a lower-environmental impact, to what extent would you support or oppose this?”

- Strongly support
- Support
- Somewhat support
- Neither support nor oppose
- Somewhat oppose
- Oppose
- Strongly oppose

1. “If supermarkets were to offer a greater range of plant-based and vegetarian products, to what extent would you support or oppose this?”

- Strongly support
- Support
- Somewhat support
- Neither support nor oppose
- Somewhat oppose
- Oppose
- Strongly oppose

1. “If supermarkets were to offer a smaller range of products with a higher-environmental impact, to what extent would you support or oppose this?”

- Strongly support
- Support
- Somewhat support
- Neither support nor oppose
- Somewhat oppose
- Oppose
- Strongly oppose

1. “If supermarkets were to offer a smaller range of meat, fish and dairy products, to what extent would you support or oppose this?”

- Strongly support
- Support
- Somewhat support
- Neither support nor oppose
- Somewhat oppose
- Oppose
- Strongly oppose

# Appendix B: Examples of product pages in the online supermarket

The screenshots below show the first 12 products of product shelves for sandwiches, pies, and ready meals. Up to 28 products can be displayed on one page.

## Figure B1. Sandwiches: Pasties & Snacking

**Control Availability only**


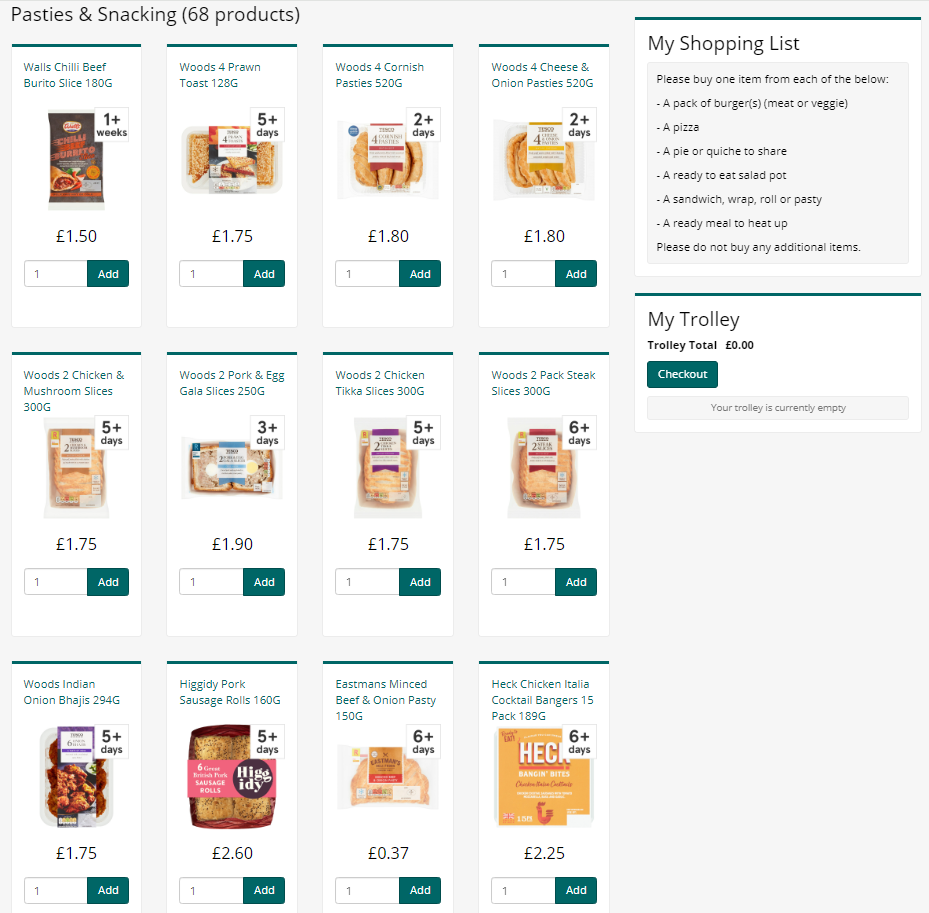

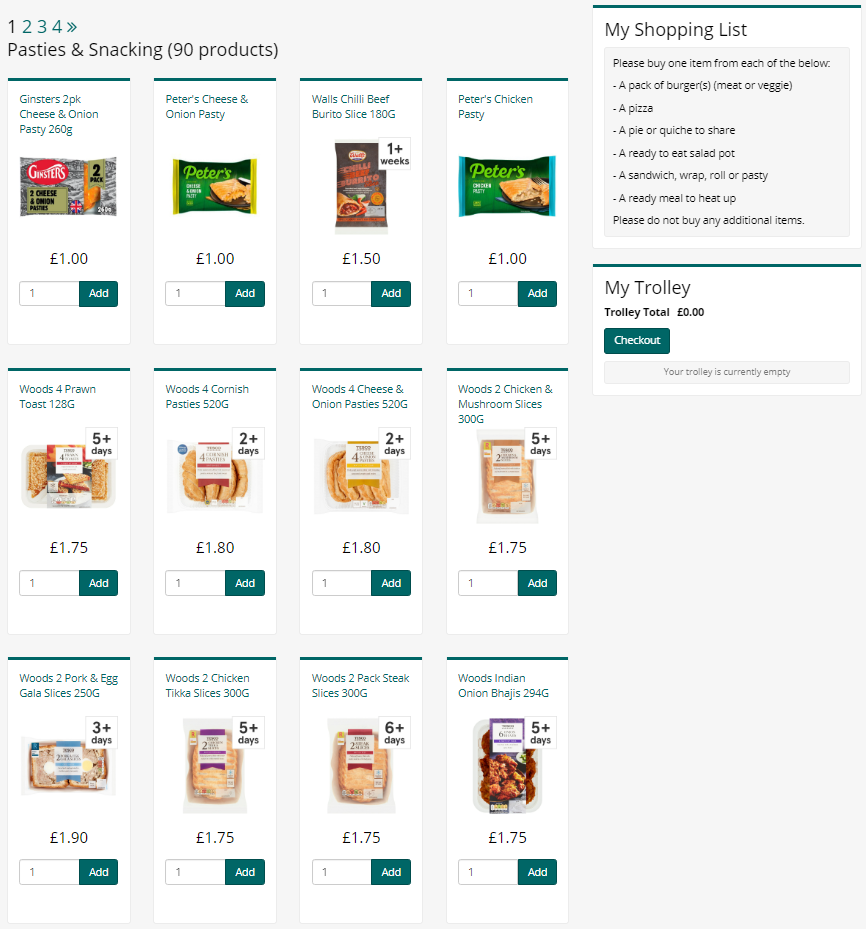


**Order only Availability & order**


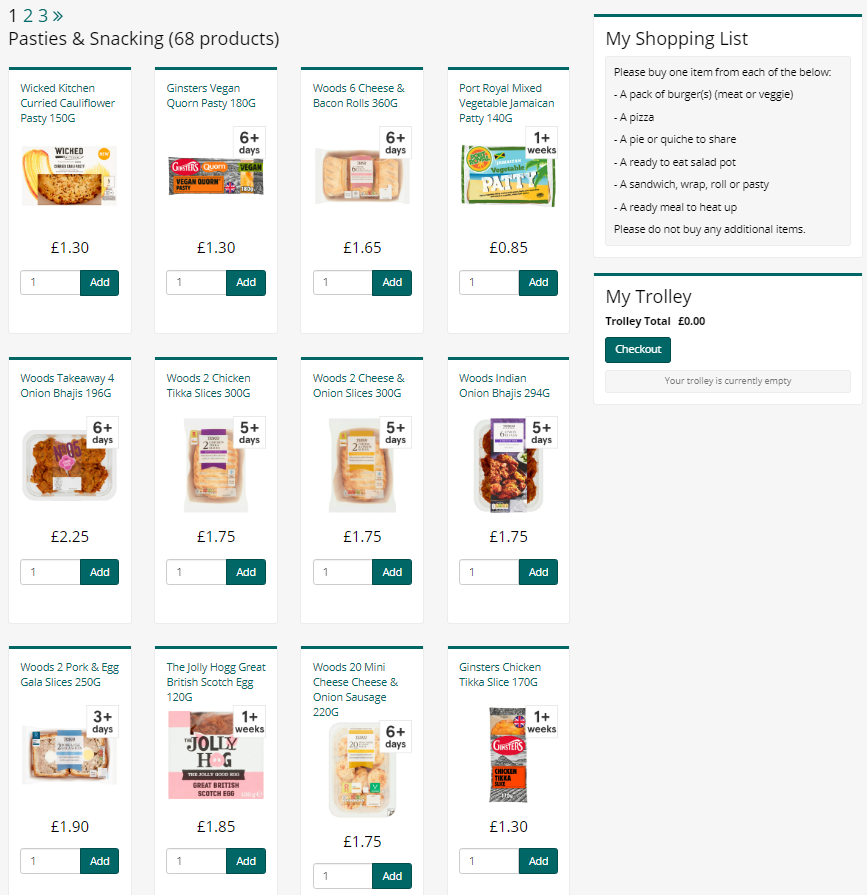

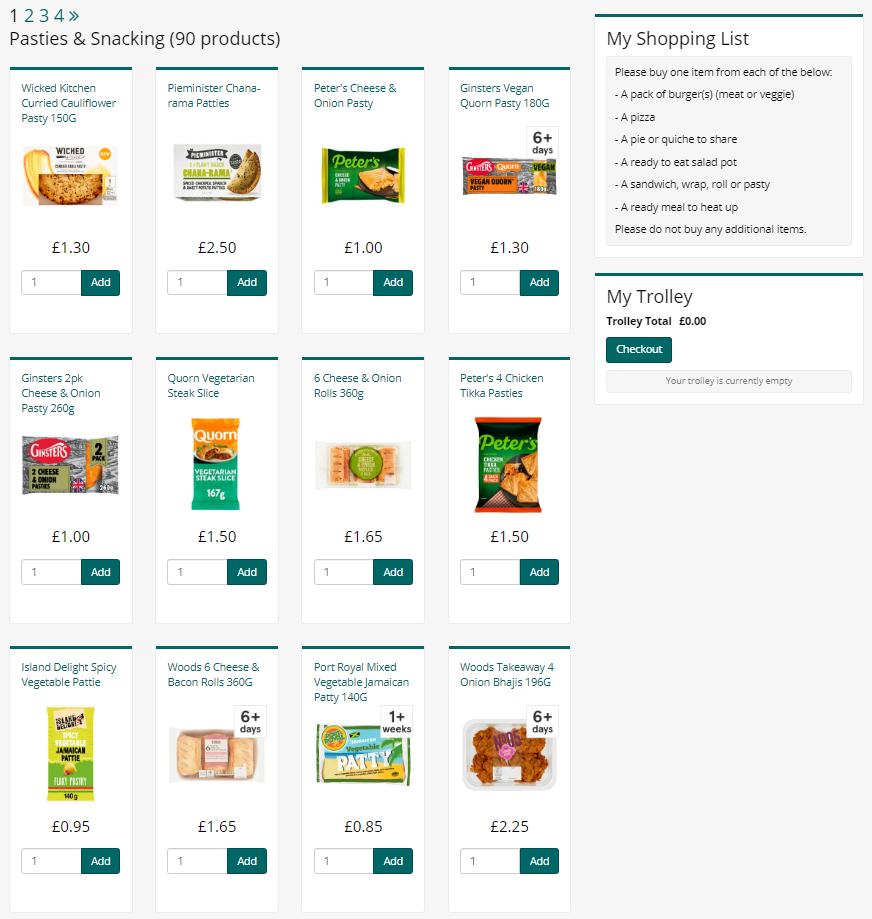


## Figure B2. Pies: All Frozen Pies

**Control Availability only**


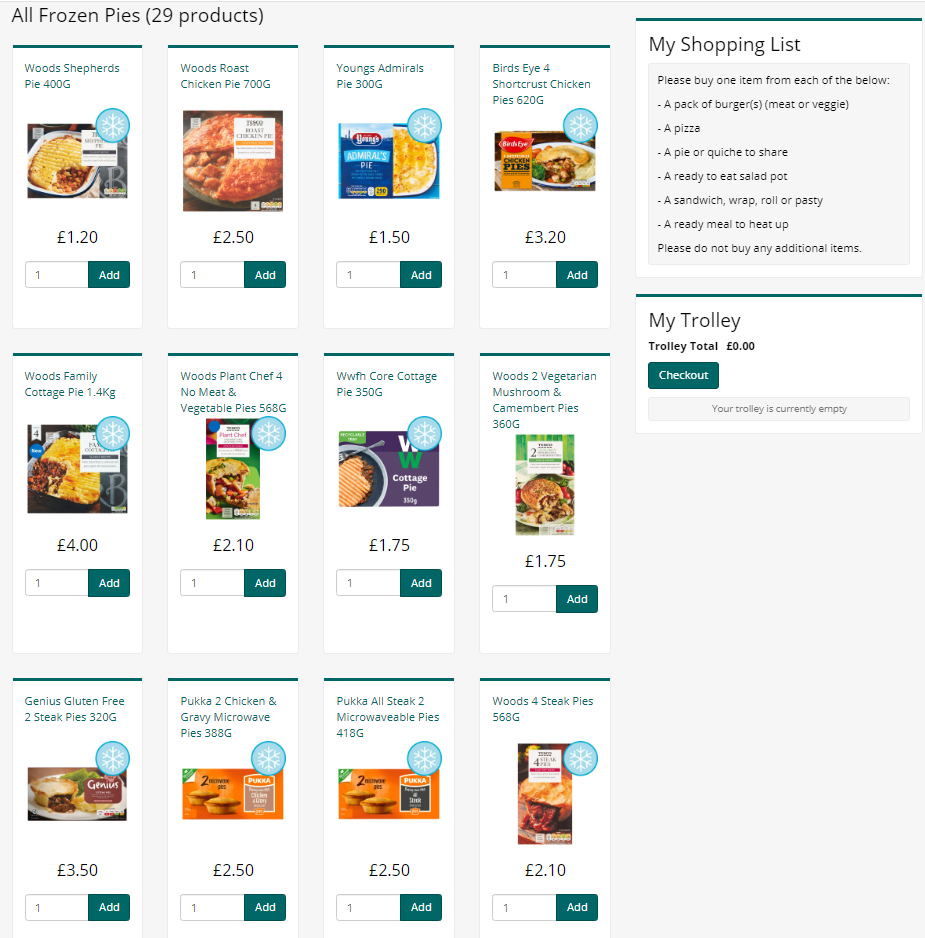

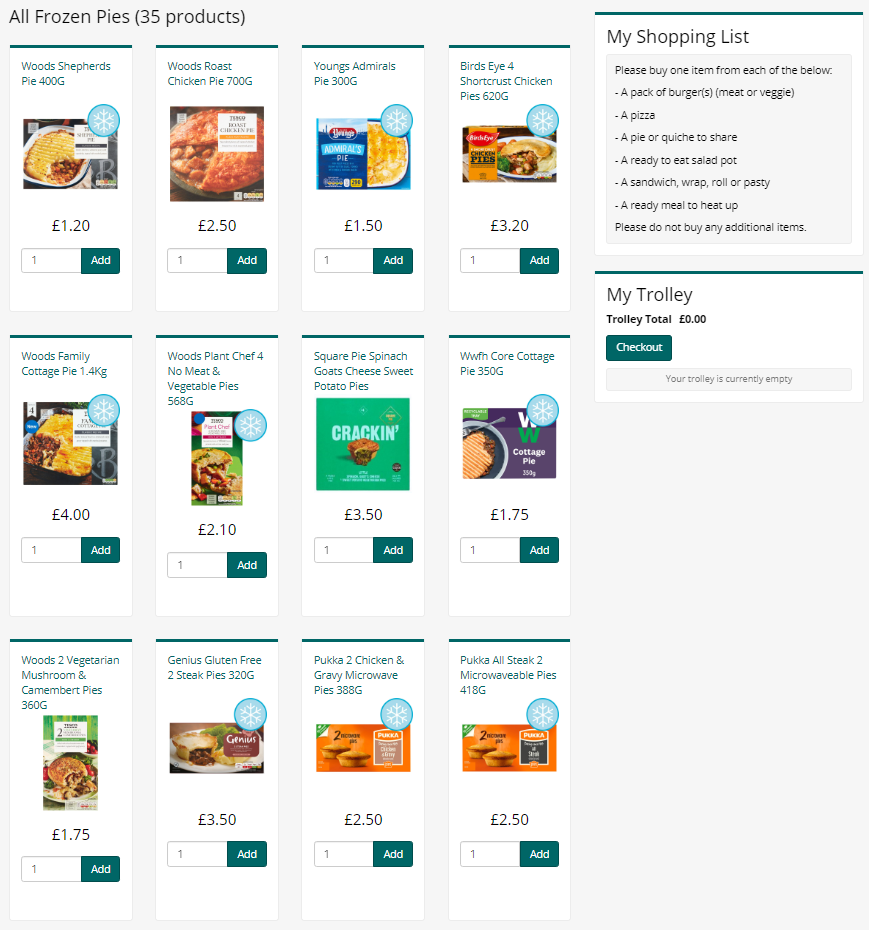


**Order only Availability & order**


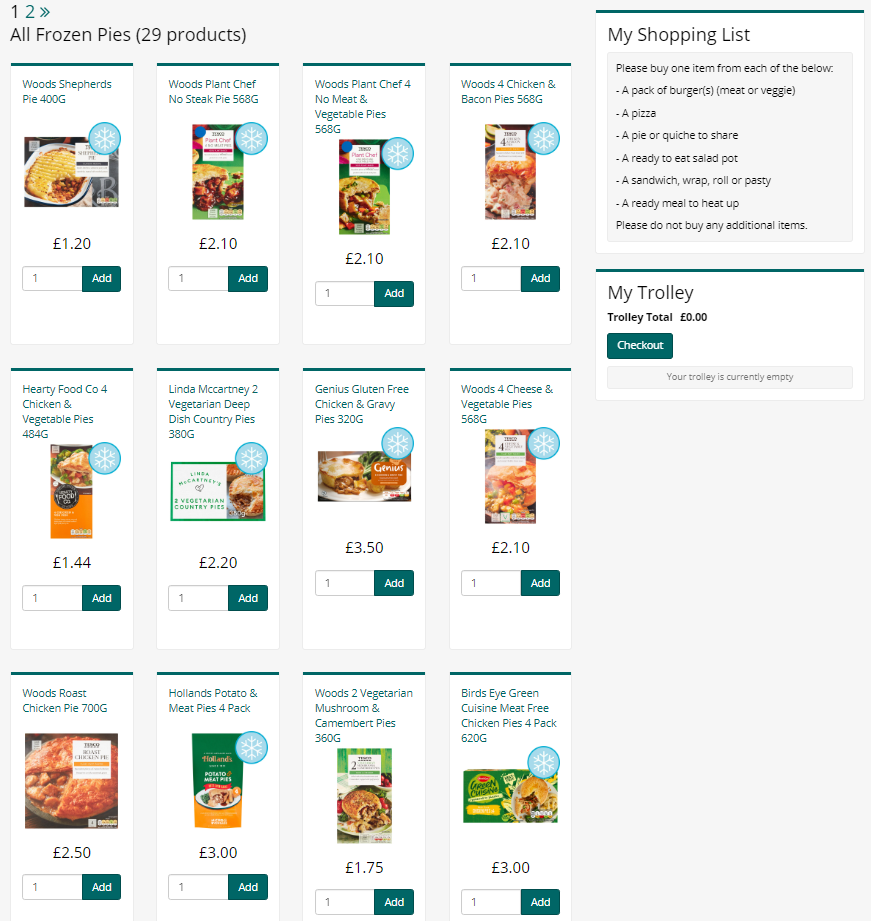

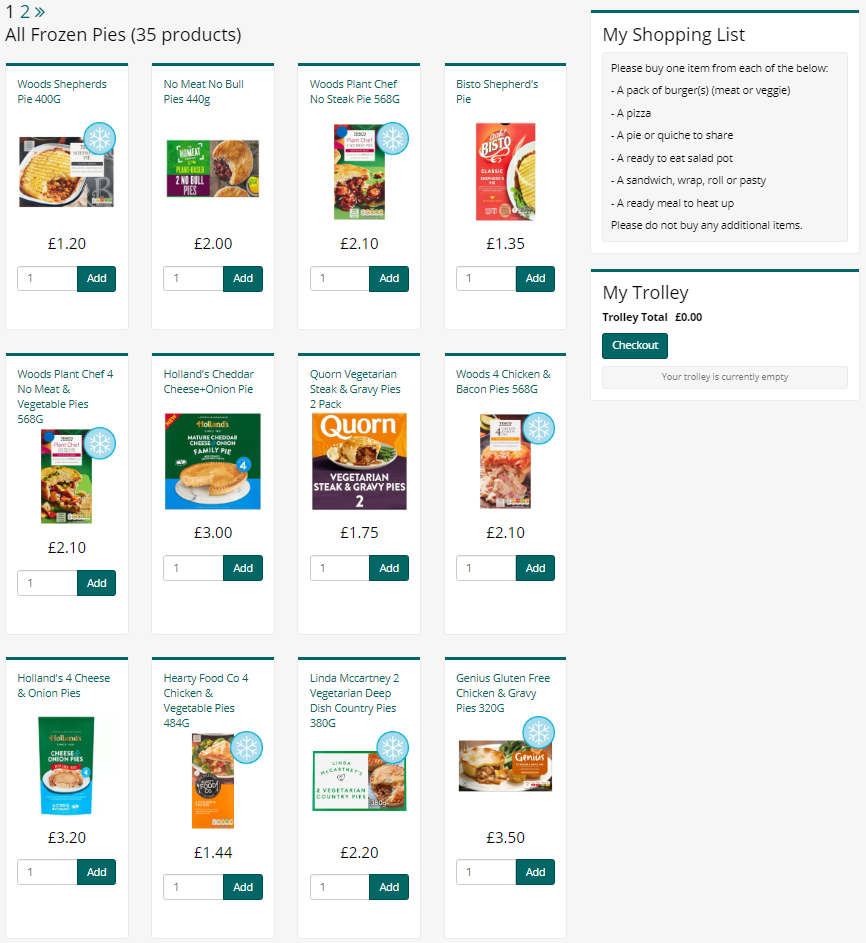


## Figure B3. Ready meals: All Ready Meals

**Control Availability only**


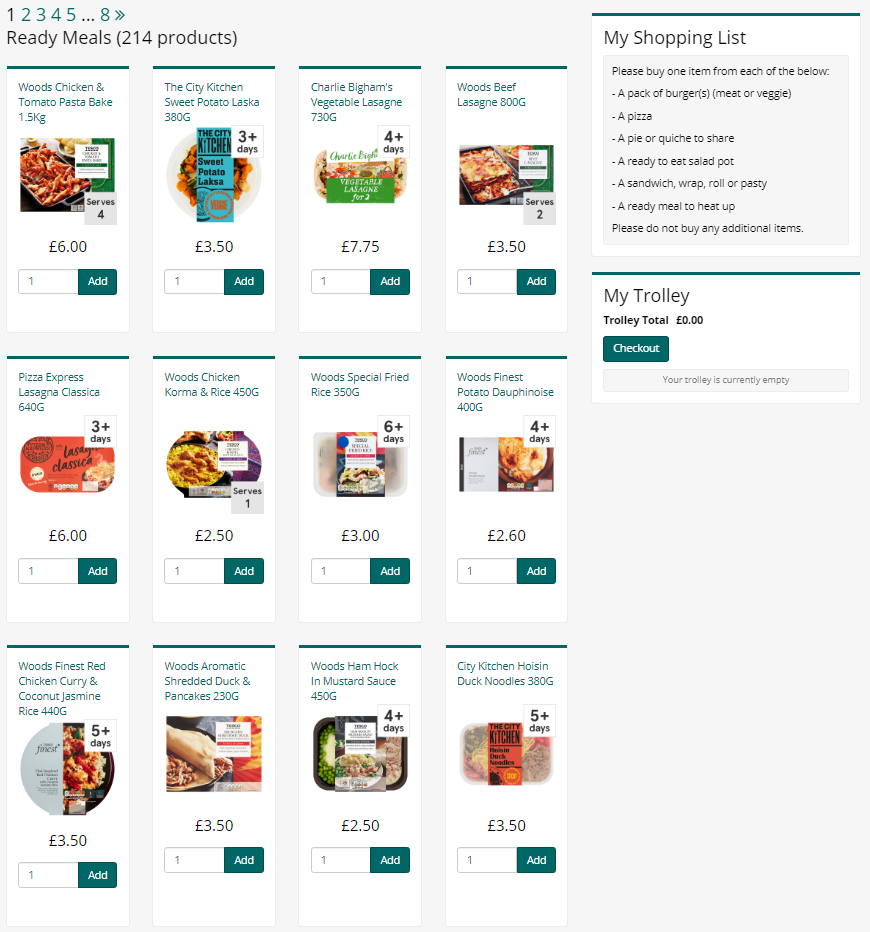

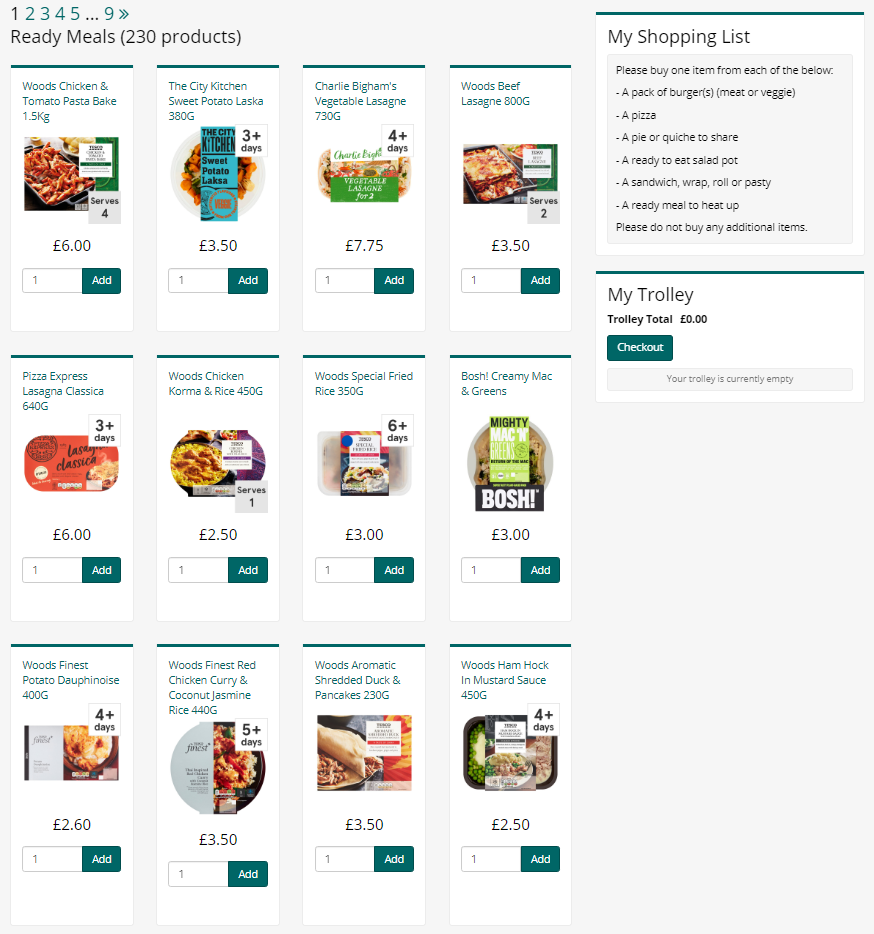


**Order only Availability & order**


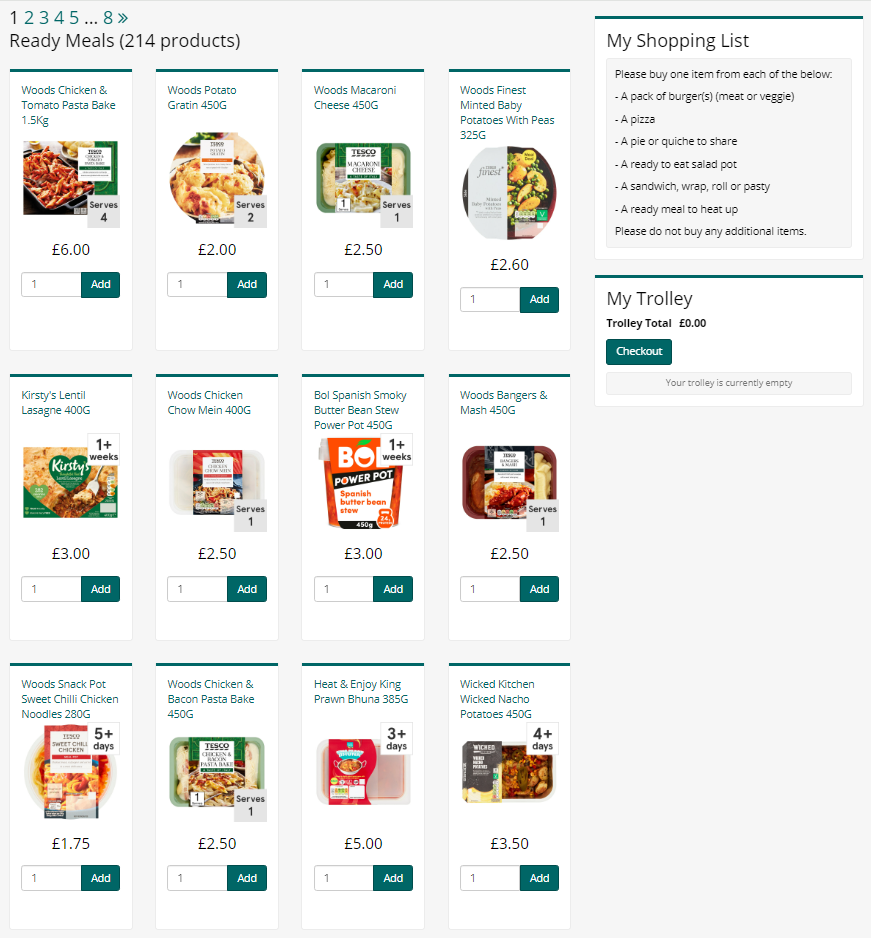

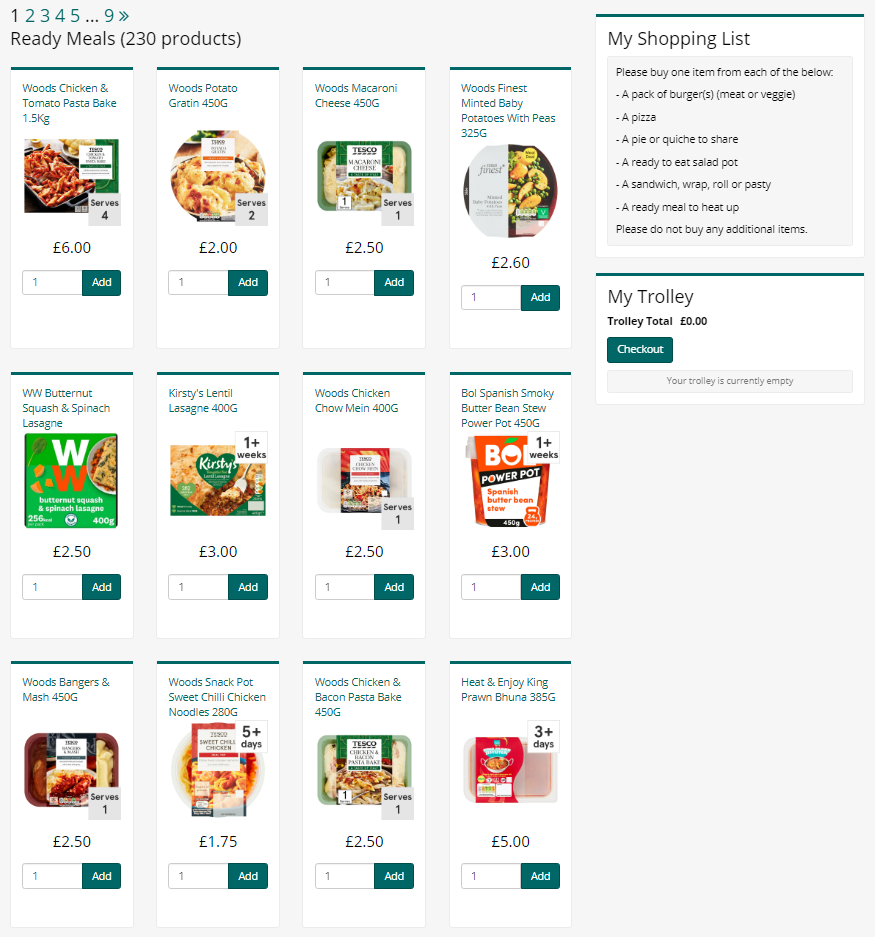


# Appendix C: Boxplot of originally planned outcome measure (ecosores) by group and analysis with burger category removed

## Figure C. Total Ecoscores of participants’ shopping baskets by group


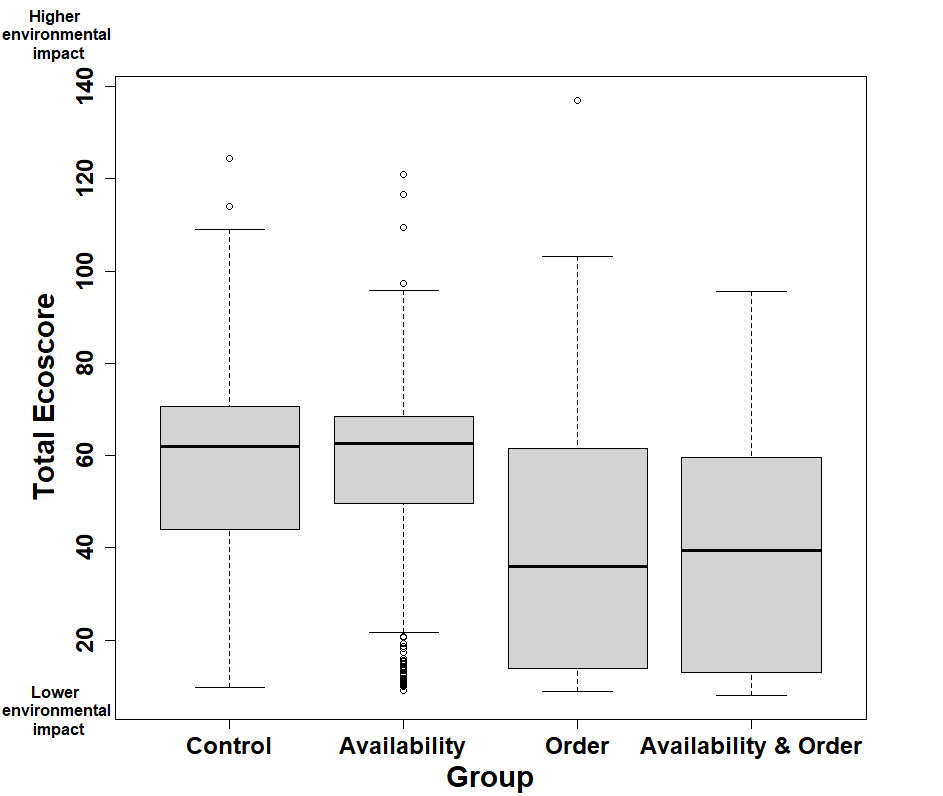


Includes data from 943 participants, according to original assigned groups.

## Table C. Total environmental impact (original Ecoscore measure) of shopping baskets between groups, with burger category removed

| Group 1 | Group 2 | Estimate | Conf. low | Conf. high | p-value |
| --- | --- | --- | --- | --- | --- |
| C | A | -0.07 | -0.17 | 0.04 | 0.332 |
| C | O | -0.17 | -0.27 | -0.07 | <0.001 (*) |
| C | AO | -0.27 | -0.38 | -0.17 | <0.001 (*) |
| A | O | -0.10 | -0.21 | 0.00 | 0.058 |
| A | AO | -0.21 | -0.31 | -0.10 | <0.001 (*) |
| O | AO | -0.10 | -0.21 | 0.00 | 0.047 (*) |

Note. (*) denotes significance at p≤0.05. Summed ecoscore variable was log transformed.

# Appendix D: Difference in proportion of low- and high-impact products selected by group (secondary outcome)

For this outcome, pre-specified multi-level logistic regression was replaced by logistic regression models for each shopping list item of interest. The mean eco quintile score of selected products was used if participants selected more than one product of the same category.

Results for burgers and ready meals were similar to the primary results (Tables D1-D4). In contrast, for pies, only the availability & order group but not the order only group had significantly higher odds of choosing a lower-impact item (OR: 3.04; 95%CI: 1.20; 4.68) and significantly lower odds of selecting a higher-impact item (OR: 0.5; 95%CI: 0.34; 0.73). Furthermore, there were no significant differences in the odds of choosing a lower-impact product for the sandwich group between the groups, whilst only the availability & order group had significantly lower odds of choosing a higher-impact item (OR: 0.48; 95%CI: 0.32; 0.71).

## Table D1: Choice of shopping list items of interest in lowest 40% of environmental impact by group

|  |  | **Estimate** | **Standard Error** | **z value** | **p-value** | **Lower 95% CI** | **Upper 95% CI** |
| --- | --- | --- | --- | --- | --- | --- | --- |
| **Burger** | *Intercept* | -2.44 | 0.24 | -10.18 |  | -2.94 | -1.99 |
|  | A | 0.34 | 0.32 | 1.04 | 0.298 | -0.29 | 0.98 |
|  | O | 1.56 | 0.28 | 5.57 | <0.001 (*) | 1.03 | 2.13 |
|  | AO | 1.49 | 0.28 | 5.29 | <0.001 (*) | 0.96 | 2.07 |
| **Sandwich** | *Intercept* | -1.76 | 0.19 | -9.20 |  | -2.15 | -1.40 |
|  | A | 0.45 | 0.26 | 1.73 | 0.083 | -0.06 | 0.96 |
|  | O | 0.41 | 0.25 | 1.63 | 0.103 | -0.08 | 0.92 |
|  | AO | 0.66 | 0.25 | 2.64 | 0.008 | 0.17 | 1.15 |
| **Pie** | *Intercept* | -1.55 | 0.17 | -9.09 |  | -1.89 | -1.22 |
|  | A | 0.44 | 0.23 | 1.91 | 0.056 | 0.01 | 0.9 |
|  | O | 0.30 | 0.23 | 1.28 | 0.200 | -0.15 | 0.75 |
|  | AO | 1.11 | 0.22 | 5.11 | <0.001 (*) | 0.69 | 1.54 |
| **Ready**  **meal** | *Intercept* | -2.93 | 0.30 | -9.88 |  | -3.56 | -2.39 |
|  | A | 0.45 | 0.39 | 1.15 | 0.251 | -0.31 | 1.23 |
|  | O | 1.88 | 0.33 | 5.67 | <0.001 (*) | 1.26 | 2.57 |
|  | AO | 2.12 | 0.33 | 6.42 | <0.001 (*) | 1.51 | 2.81 |

## Table D2: Odds ratio and percentage of participants choosing a lower-impact item

|  |  | **OR** | **Lower 95% CI** | **Upper 95% CI** | **%*** |
| --- | --- | --- | --- | --- | --- |
| **Burger** | *Intercept* |  |  |  | 8% |
|  | A | 1.4 | 0.75 | 2.66 | 11% |
|  | O | 4.74 | 2.79 | 8.39 | 29% |
|  | AO | 4.45 | 2.61 | 7.93 | 28% |
| **Sandwich** | *Intercept* |  |  |  | 15% |
|  | A | 1.56 | 0.95 | 2.60 | 21% |
|  | O | 1.51 | 0.92 | 2.50 | 21% |
|  | AO | 1.93 | 1.19 | 3.16 | 25% |
| **Pie** | *Intercept* |  |  |  | 18% |
|  | A | 1.55 | 0.99 | 2.45 | 25% |
|  | O | 1.34 | 0.86 | 2.12 | 22% |
|  | AO | 3.04 | 2.00 | 4.68 | 39% |
| **Ready**  **meal** | *Intercept* |  |  |  | 5% |
|  | A | 1.56 | 0.73 | 3.43 | 8% |
|  | O | 6.54 | 3.54 | 13.11 | 26% |
|  | AO | 8.31 | 4.51 | 16.60 | 31% |

*% = percentage of participants choosing a lower-impact item.

Number of participants: burgers=909 [C: 236; A: 220; O: 232; AO: 221], sandwiches=857 [C: 218; A: 203; O: 223; AO: 213], pies=927 [C: 239; A: 221; O: 238; AO: 229], ready meals=915 [C: 236; A: 220; O: 235; AO: 224]

Due to several tests being conducted, exploratory analyses applied the same significance threshold as secondary analyses (p≤0.003).

## Table D3: Choice of shopping list items of interest in highest 40% of environmental impact by group

|  |  | **Estimate** | **Standard Error** | **z value** | **p-value** | **Lower 95% CI** | **Upper 95% CI** |
| --- | --- | --- | --- | --- | --- | --- | --- |
| **Burger** | *Intercept* | 1.82 | 0.19 | 9.68 |  | 1.46 | 2.20 |
|  | A | 0.03 | 0.27 | 0.11 | 0.915 | -0.50 | 0.57 |
|  | O | -1.14 | 0.23 | -4.87 | <0.001 (*) | -1.60 | -0.69 |
|  | AO | -1.21 | 0.23 | -5.16 | <0.001 (*) | -1.68 | -0.76 |
| **Sandwich** | *Intercept* | -0.11 | 0.14 | -0.81 |  | -0.38 | 0.16 |
|  | A | -0.14 | 0.20 | -0.70 | 0.483 | -0.52 | 0.25 |
|  | O | -0.49 | 0.19 | -2.51 | 0.012 | -0.87 | -0.11 |
|  | AO | -0.73 | 0.20 | -3.64 | <0.001 (*) | -1.13 | -0.34 |
| **Pie** | *Intercept* | -0.09 | 0.13 | -0.71 |  | -0.35 | 0.16 |
|  | A | -0.14 | 0.19 | -0.72 | 0.471 | -0.50 | 0.23 |
|  | O | -0.18 | 0.18 | -0.97 | 0.332 | -0.54 | 0.18 |
|  | AO | -0.69 | 0.19 | -3.57 | <0.001 (*) | -1.07 | -0.31 |
| **Ready**  **meal** | *Intercept* | 0.78 | 0.14 | 5.58 |  | 0.51 | 1.06 |
|  | A | 0.06 | 0.20 | 0.31 | 0.754 | -0.33 | 0.46 |
|  | O | -0.84 | 0.19 | -4.40 | <0.001 (*) | -1.22 | -0.47 |
|  | AO | -1.20 | 0.20 | -6.13 | <0.001 (*) | -1.59 | -0.82 |

## Table D4: Odds ratio and percentage of participants choosing a higher-impact item

|  |  | **OR** | **Lower 95% CI** | **Upper 95% CI** | **%*** |
| --- | --- | --- | --- | --- | --- |
| **Burger** | *Intercept* |  |  |  | 86% |
|  | A | 1.03 | 0.60 | 1.76 | 86% |
|  | O | 0.32 | 0.20 | 0.50 | 66% |
|  | AO | 0.3 | 0.19 | 0.47 | 65% |
| **Sandwich** | *Intercept* |  |  |  | 47% |
|  | A | 0.87 | 0.59 | 1.28 | 44% |
|  | O | 0.61 | 0.42 | 0.90 | 35% |
|  | AO | 0.48 | 0.32 | 0.71 | 30% |
| **Pie** | *Intercept* |  |  |  | 48% |
|  | A | 0.87 | 0.60 | 1.26 | 44% |
|  | O | 0.84 | 0.58 | 1.20 | 43% |
|  | AO | 0.5 | 0.34 | 0.73 | 31% |
| **Ready**  **meal** | *Intercept* |  |  |  | 69% |
|  | A | 1.07 | 0.72 | 1.59 | 70% |
|  | O | 0.43 | 0.29 | 0.63 | 49% |
|  | AO | 0.3 | 0.20 | 0.44 | 40% |

*% = percentage of participants choosing a higher-impact item.

Due to several tests being conducted, exploratory analyses applied the same significance threshold as secondary analyses (p≤0.003).

Number of participants: burgers=909 [C: 236; A: 220; O: 232; AO: 221], sandwiches=857 [C: 218; A: 203; O: 223; AO: 213], pies=927 [C: 239; A: 221; O: 238; AO: 229], ready meals=915 [C: 236; A: 220; O: 235; AO: 224]

# Appendix E: Effect of interventions within food categories

Instead of using linear regression as pre-specified in the protocol, chi-square tests and descriptive statistics were used with eco quintile scores treated as a categorical variable. Mean eco quintile scores of selected items were used to account for participants who selected more than one product of the same category.

A similar pattern to the primary outcome was seen for all items apart from pies, with the order only and availability & order groups having a higher share of products with an eco quintile score of 1 and 2 and a lower share of eco quintile scores 4 and 5 compared to the control and availability only groups (Figure E). For pies, the distribution of eco quintiles in the availability only and order only groups was comparable.

Significant differences were found in the proportion of eco quintile scores of products selected by participants within categories of interest between groups for burgers, pies, and ready meals (p-values: burger=<0.001; pie=<0.001; ready meals=<0.001), whilst the result for sandwiches was not significant (p=0.005; compared to our significance threshold of p=0.003). Due to some expected cell counts below 5, the chi-square test for the sandwich category was also run combining products with an eco quintile of 1 and eco quintile of 2 per group, with the result remaining insignificant (p=0.004).


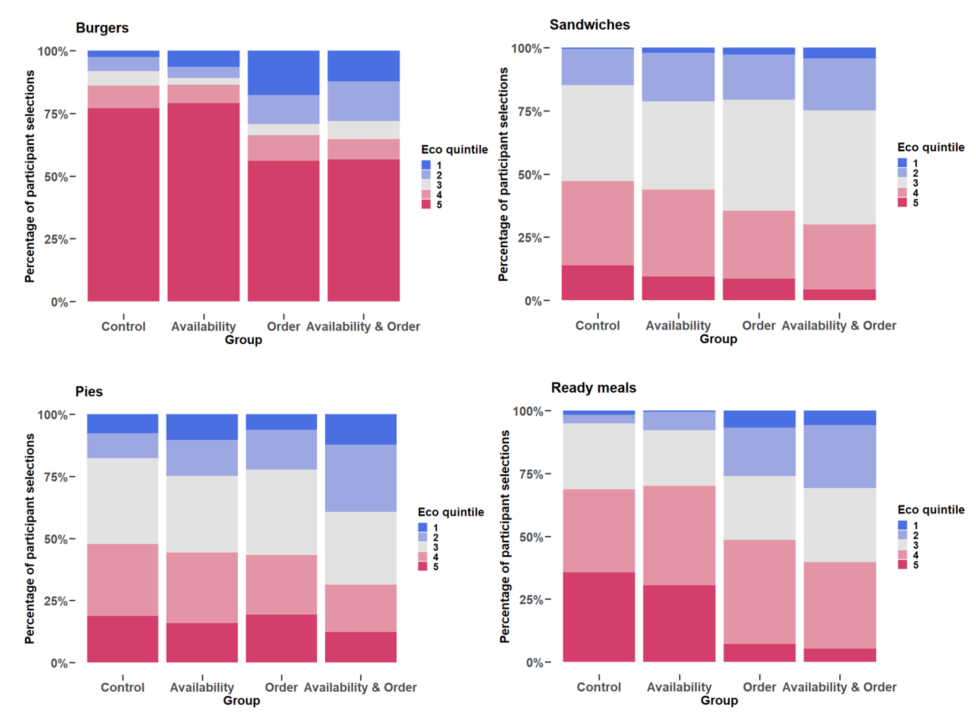


**Figure E.** Eco quintile distribution of burgers, sandwiches, pies, and ready meals selected by participants, by group. *Burgers:* X-squared=74.583; df=12; p-value=<0.001; *sandwiches:* X-squared=28.278; df=12; p-value=0.005; with eco quintiles 1&2 combined: X-squared=24.441; df=9; p-value=0.004*; pies:* X-squared=38.984; df=12; p-value=<0.001*; ready meals:* X-squared=159.048; df=12; p-value=<0.001. Number of participants: burgers=909 [C: 236; A: 220; O: 232; AO: 221], sandwiches=857 [C: 218; A: 203; O: 223; AO: 213], pies=927 [C: 239; A: 221; O: 238; AO: 229], ready meals=915 [C: 236; A: 220; O: 235; AO: 224].

# Appendix F: Sensitivity analysis

| Group 1 | Group 2 | Estimate | p-value | Conf. low | Conf. high |
| --- | --- | --- | --- | --- | --- |
| C | A | -0.06 | 0.996 | -0.82 | 0.69 |
| C | O | -1.80 | <0.001 (*) | -2.62 | -0.99 |
| C | AO | -2.25 | <0.001 (*) | -3.05 | -1.46 |
| A | O | -1.74 | <0.001(*) | -2.59 | -0.89 |
| A | AO | -2.19 | <0.001 (*) | -3.02 | -1.36 |
| O | AO | -0.45 | 0.554 | -1.34 | 0.43 |

Includes data from 662 participants, according to original assigned groups [C: 170; A: 152; O: 172; AO: 168].

# Appendix G: Analysis of individual environmental indicators

| Indicator | Group | Estimate | Standard error | t-value | p-value | Lower 95% CI | Upper 95% CI |
| --- | --- | --- | --- | --- | --- | --- | --- |
| GHG | Intercept | 22.00 | 0.20 | 107.36 |  | 21.59 | 22.40 |
|  | A | 0.09 | 0.29 | 0.31 | 0.755 | -0.49 | 0.67 |
|  | O | -1.29 | 0.29 | -4.46 | <0.001 (*) | -1.86 | -0.73 |
|  | AO | -1.85 | 0.29 | -6.31 | <0.001 (*) | -2.42 | -1.27 |
| Water scarcity | Intercept | 22.79 | 0.20 | 112.46 |  | 22.39 | 23.19 |
|  | A | -0.02 | 0.29 | -0.07 | 0.941 | -0.59 | 0.55 |
|  | O | -1.34 | 0.29 | -4.66 | <0.001 (*) | -1.90 | -0.77 |
|  | AO | -2.05 | 0.29 | -7.07 | <0.001 (*) | -2.62 | -1.48 |
| Land  use | Intercept | 20.96 | 0.19 | 112.77 |  | 20.59 | 21.32 |
|  | A | -0.05 | 0.27 | -0.19 | 0.849 | -0.57 | 0.47 |
|  | O | -1.12 | 0.26 | -4.24 | <0.001 (*) | -1.63 | -0.60 |
|  | AO | -1.43 | 0.27 | -5.38 | <0.001 (*) | -1.95 | -0.91 |
| Eutrophi-cation | Intercept | 21.40 | 0.22 | 98.10 |  | 20.98 | 21.83 |
|  | A | 0.14 | 0.31 | 0.46 | 0.648 | -0.47 | 0.76 |
|  | O | -1.26 | 0.31 | -4.06 | <0.001 (*) | -1.86 | -0.65 |
|  | AO | -1.78 | 0.31 | -5.69 | <0.001 (*) | -2.39 | -1.16 |

Includes data from 943 participants, according to original assigned groups. Control group was the reference group.

Secondary analyses applied a threshold of p≤0.003 (Bonferroni adjustment) to determine statistical significance. GHG = greenhouse gas emissions.

# Appendix H: Total Eco quintile score of participant’s shopping baskets by group, demographic characteristics and device type used for shopping online

| **Variable** | | **Estimate** | **Standard Error** | **t value** | **p-value** | **Lower 95% CI** | **Upper 95% CI** |
| --- | --- | --- | --- | --- | --- | --- | --- |
| **Intercept** | | 20.31 | 0.56 | 36.38 |  | 19.21 | 21.40 |
| **Group** | |  |  |  |  |  |  |
| *C* (ref) | |  |  |  |  |  |  |
|  | *A* | -0.02 | 0.30 | -0.07 | 0.95 | -0.60 | 0.56 |
|  | *O* | -1.72 | 0.29 | -5.89 | <0.001 (*) | -2.29 | -1.15 |
|  | *AO* | -2.32 | 0.29 | -7.88 | <0.001 (*) | -2.90 | -1.74 |
| **Gender** | |  |  |  |  |  |  |
|  | *Female (ref)* |  |  |  |  |  |  |
|  | *Male* | 1.70 | 0.38 | 4.42 | 0.15 | 0.95 | 2.45 |
|  | *Identify as another gender* | -0.33 | 0.23 | -1.43 | 0.77 | -0.78 | 0.12 |
| **Age group** | |  |  |  |  |  |  |
|  | *18-24 (ref)* |  |  |  |  |  |  |
|  | *25-34* | 0.40 | 0.39 | 1.03 | 0.30 | -0.36 | 1.16 |
|  | *35-44* | 0.61 | 0.40 | 1.54 | 0.12 | -0.17 | 1.39 |
|  | *45-54* | 1.29 | 0.39 | 3.33 | <0.001 (*) | 0.53 | 2.05 |
|  | *55-64* | 1.36 | 0.40 | 3.38 | <0.001 (*) | 0.57 | 2.15 |
|  | *65+* | 1.70 | 0.38 | 4.42 | <0.001 (*) | 0.95 | 2.45 |
| **Education** | |  |  |  |  |  |  |
|  | *Up to 4 GCSE’s (ref)* |  |  |  |  |  |  |
|  | *5 or more GCSE’s or 1 A-level* | 0.27 | 0.42 | 0.63 | 0.53 | -0.56 | 1.09 |
|  | *2 or more A-levels* | -0.05 | 0.40 | -0.11 | 0.91 | -0.82 | 0.73 |
|  | *Bachelor’s degree* | -0.61 | 0.38 | -1.60 | 0.11 | -1.36 | 0.14 |
|  | *Postgraduate degree* | -0.77 | 0.42 | -1.81 | 0.07 | -1.60 | 0.06 |
| **Income** | |  |  |  |  |  |  |
|  | *Below £15.5k (ref)* |  |  |  |  |  |  |
|  | *Between £15.5k up to and including £25k* | 0.69 | 0.37 | 1.85 | 0.06 | -0.04 | 1.42 |
|  | *Between £25k and £39k* | 0.57 | 0.35 | 1.64 | 0.10 | -0.11 | 1.25 |
|  | *£40k or above* | 0.64 | 0.33 | 1.91 | 0.06 | -0.02 | 1.29 |
|  | *Prefer not to say* | 0.32 | 0.54 | 0.59 | 0.55 | -0.74 | 1.38 |
| **Meat consumption** | |  |  |  |  |  |  |
|  | *Low (ref)* |  |  |  |  |  |  |
|  | *Medium* | 0.73 | 0.25 | 2.95 | 0.003 | 0.25 | 1.22 |
|  | *High* | 0.30 | 0.28 | 1.07 | 0.29 | -0.25 | 0.84 |
| **Device used for study** | |  |  |  |  |  |  |
|  | *Desktop/Laptop (ref)* |  |  |  |  |  |  |
|  | *Mobile phone/Tablet* | -0.06 | 0.23 | -0.24 | 0.81 | -0.51 | 0.40 |

Included 937 participants according to original assigned groups (C: 241; A: 226; O: 239; AO: 231), 6 participants dropped due to not indicating their meat consumption.

Secondary analyses applied a threshold of p≤0.003 (Bonferroni adjustment) to determine statistical significance.

Education categories adapted from UK census categories ^(1)^: None; Up to 4 GCSE's (Including 1-4 O Levels/CSE/GCSEs (any grades), Foundation Diploma, NVQ level 1, Foundation GNVQ or equivalents); 5 or more GCSE's or 1 A-level (Including 5+ GCSEs (Grades A*-C),1 A Level/ 2-3 AS Levels, NVQ level 2, Intermediate GNVQ, City and Guilds Craft, BTEC First/General Diploma, RSA Diploma, Apprenticeship or equivalents); 2 or more A-levels (Including 2+ A Levels, 4+ AS Levels, NVQ Level 3, Advanced GNVQ, City and Guilds Advanced Craft, ONC, OND, BTEC National, RSA Advanced Diploma or equivalents); Bachelor's degree (Including BA, BSc, NVQ Level 4-5, HNC, HND, RSA Higher Diploma, BTEC Higher level or equivalents); Post-Graduate degree or qualification (Including Higher Degrees e.g. MA, PhD, PGCE, Professional qualifications e.g. teaching, nursing, accountancy or equivalents). Category “None” combined in analysis with “Up to 4 GCSE's”.

# Appendix I: Acceptability of the interventions

## Figure I1. Acceptability of the positioning intervention


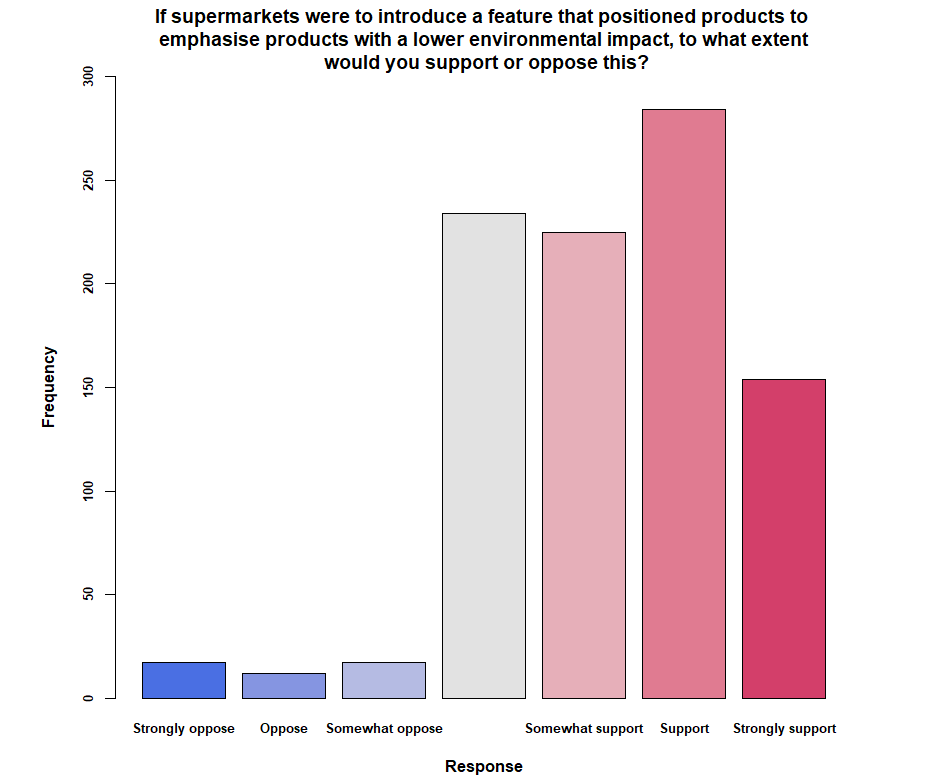


Includes 943 participants

## Figure I2. Acceptability of increasing the availability of lower-impact products


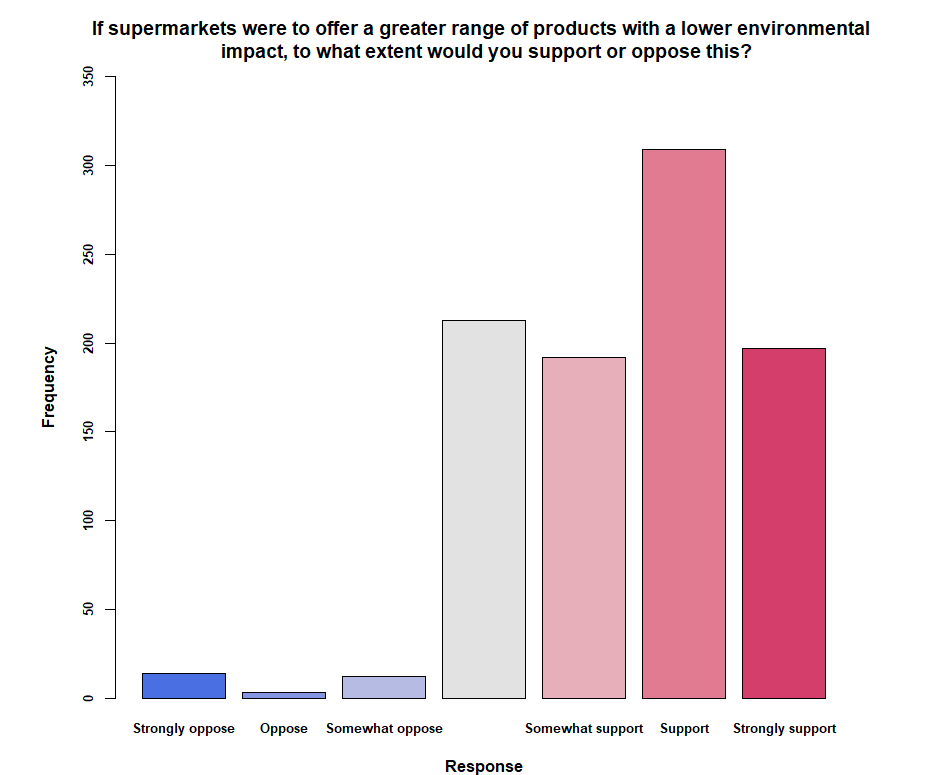


Includes 940 participants [C: 241; A: 227; O: 240; AO: 232].

## Figure I3. Acceptability of increasing the availability of vegetarian and plant-based products


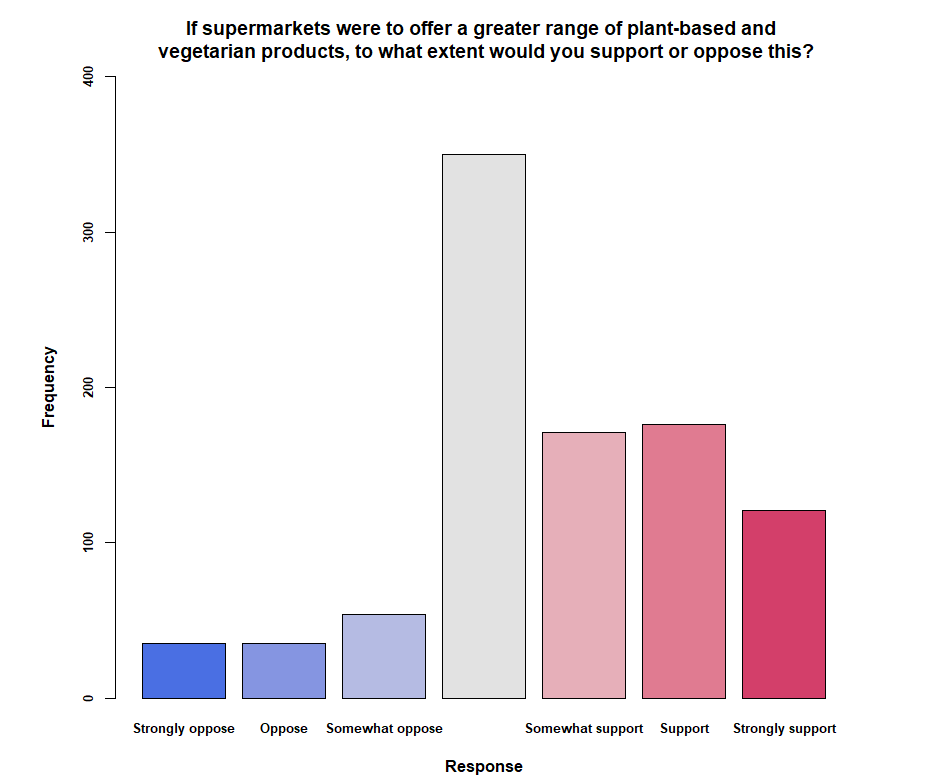


Includes 942 participants [C: 242; A: 227; O: 241; AO: 232].

## Figure I4. Acceptability of decreasing the availability of higher-impact products


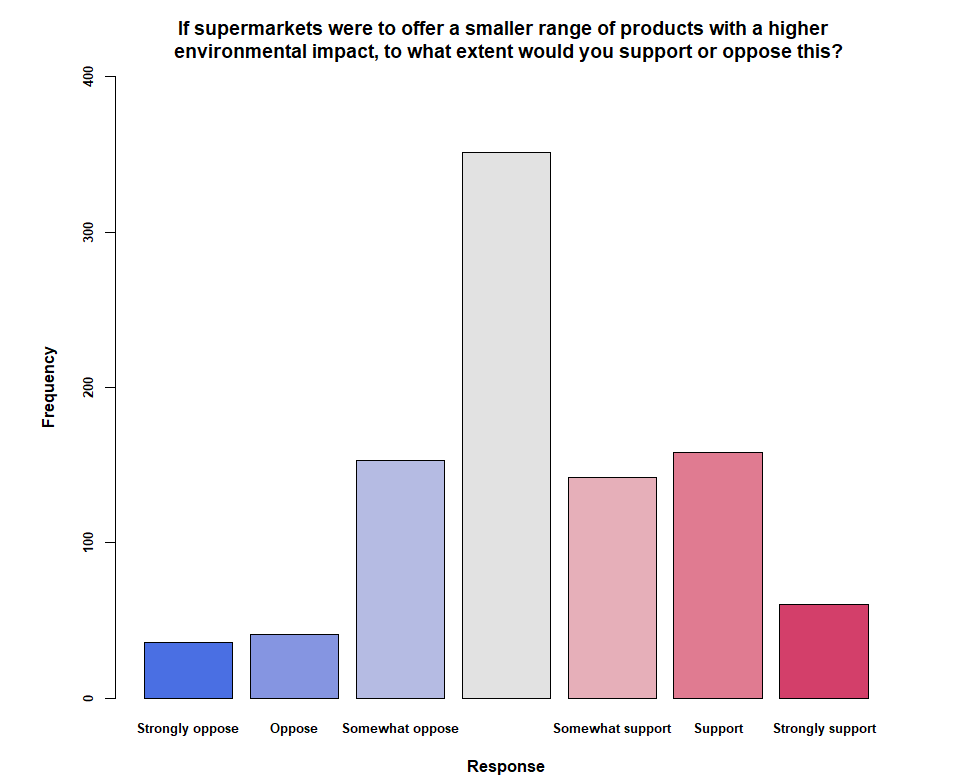


Includes 941 participants [C: 242; A: 227; O: 240; AO: 232].

## Figure I5. Acceptability of decreasing the availability of meat, fish and dairy products


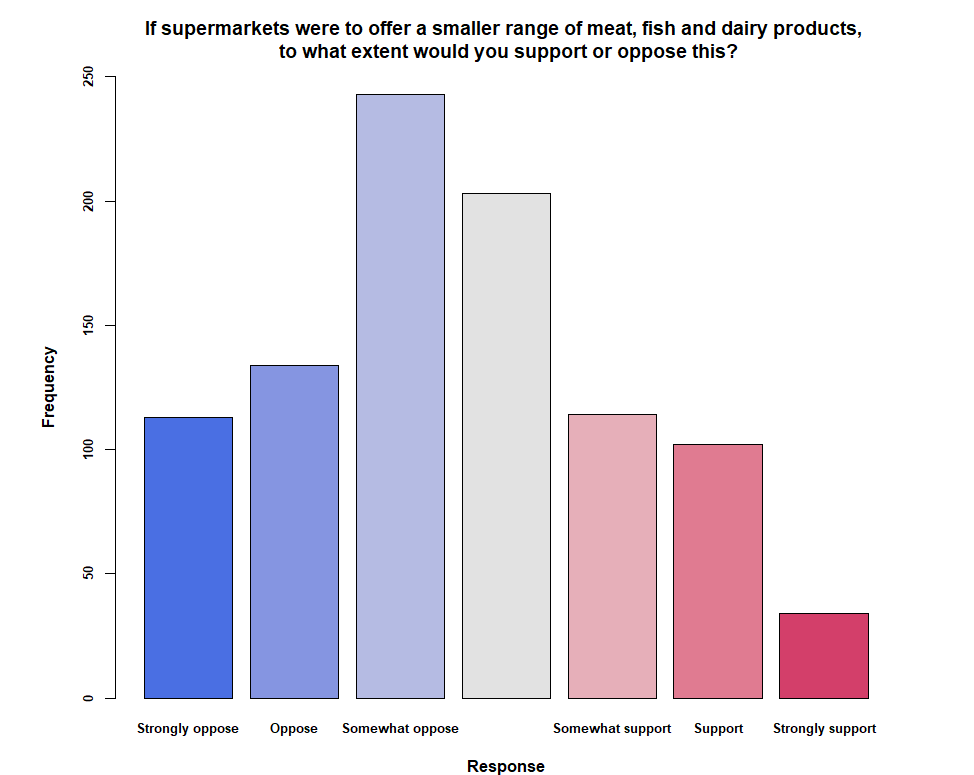


Includes 943 participants.

# Appendix J: Welch ANOVA for basket price

| y | n | Statistic | DFn | p-value |
| --- | --- | --- | --- | --- |
| basketprice | 943 | 2.61 | 3 | 0.05 |

Includes 943 participants according to original assigned groups.

Welch ANOVA was run due to unequal variances (Bartlett test: p=0.001329). Due to several tests being conducted, exploratory analyses applied the same significance threshold as secondary analyses (p≤0.003).

# References

1. Office for National Statistics (ONS). 2011 Census: Key Statistics and Quick Statistics for Local Authorities in the United Kingdom 2013 [cited 2023 7 Sep]. Available from: <https://www.ons.gov.uk/employmentandlabourmarket/peopleinwork/employmentandemployeetypes/bulletins/keystatisticsandquickstatisticsforlocalauthoritiesintheunitedkingdom/2013-12-04#qualifications>.
